# Perinatal short-chain fructo-oligosaccharide supplementation to sow affects colostrum quality, and consequently microbiota composition and performances of progeny

**DOI:** 10.1093/jas/skag140

**Published:** 2026-05-09

**Authors:** Cindy Le Bourgot, Jeroen Degroote, Agathe Roméo, Maryam Majdeddin, Noémie Van Noten, Elout Van Liefferinge, Joris Michiels

**Affiliations:** Scientific & Regulatory Affairs, Tereos, Moussy-Le-Vieux, 77290, France; Laboratory for Animal Nutrition and Animal Product Quality (LANUPRO), Ghent University, Gent, B-9000, Belgium; Scientific & Regulatory Affairs, Tereos, Moussy-Le-Vieux, 77290, France; Laboratory for Animal Nutrition and Animal Product Quality (LANUPRO), Ghent University, Gent, B-9000, Belgium; Laboratory for Animal Nutrition and Animal Product Quality (LANUPRO), Ghent University, Gent, B-9000, Belgium; Laboratory for Animal Nutrition and Animal Product Quality (LANUPRO), Ghent University, Gent, B-9000, Belgium; Laboratory for Animal Nutrition and Animal Product Quality (LANUPRO), Ghent University, Gent, B-9000, Belgium

**Keywords:** prebiotic, short-chain fructo-oligosaccharides, intestinal microbiota, immune transfer, growth

## Abstract

Perinatal nutrition of the sow plays a decisive role in controlling gut bacterial primo-colonization in the offspring and, thereby, is an important driver of the development of piglets. Since short-chain fructo-oligosaccharides (scFOS) are known to positively modulate the gut microbiota composition and its fermentative activity, our study aimed at evaluating the effects of such supplementation to the sow during the perinatal period on nutritional and immune quality of colostrum and milk, and gut microbiota establishment, and subsequent consequences on growth performances of piglets. Sows received a control diet or a diet supplemented with either a low dose (Low scFOS) or moderate dose of scFOS (Mid scFOS): 0.15% or 0.33% from d28 pre-partum to d2 post-partum, then 0.10% or 0.15% until the end of the lactation period, respectively. At 23 d of age, piglets were weaned on a standard diet and followed for growth parameters until 67 d of age. The supplementation of scFOS in sow diet improved the nutritional composition of colostrum and its immune quality by increasing protein content (Low and Mid scFOS), IgG (Low scFOS) and TGF-β1 (Mid scFOS) concentrations. Few changes were recorded regarding the sow fecal microbiota composition while the short-chain fatty acids (SCFA) synthesis was significantly increased in Low scFOS group, with Mid scFOS group being intermediate. On the contrary, in neonates, significant changes in fecal microbiota composition were measured, while SCFA production was not different between groups. Some strains of *Lactobacillus* were greatly increased upon maternal scFOS supplementation. The modulation of the microbiota resulted in a significant reduction of feed conversion ratio in weaned pigs born from scFOS supplemented mothers. Our results demonstrated that scFOS addition in the maternal diet represents a beneficial nutritional strategy to improve the quality of colostrum and modulate the gut microbiota colonization, positively impacting feed efficiency of piglets.

## Teaser

Adding short-chain fructo-oligosaccharides to the diet of sows during the perinatal period improves the growth performance of their piglets, through the modulation of their gut microbiota.

## Introduction

Prenatal and early postnatal life represent critical periods of development for the piglet during which different systems become established, such as the gut microbiota, intestinal functions, and its immune system. Nutrition plays a decisive role during this perinatal period, acting both on the mother and her offspring.

From birth, the immaturity of the newborn’s immune system is partially compensated by the acquisition of passive immunity provided by colostrum and milk. Colostrum provides energy to the newborn, many growth factors as well as immune protection with the presence of specific maternal immunoglobulins like IgG and IgA, and immune cells (Harris et al. 2006). Indeed, colostrum is rich in IgG (systemic immunity), and then milk provides IgM and especially IgA essential for the defense of intestinal mucous membranes (mucosal immunity). This passive transfer of immunoglobulins is the first line of defense of the newborn against various pathogenic bacteria and some viruses to ensure its growth and survival after birth, while the neonatal immune system continues to develop before becoming fully functional (Salmon et al. 2009). Colostrum and milk also contain other immunomodulatory factors such as TGF-β1, an immunoregulatory cytokine playing an essential role in the newborn, especially in terms of induction of oral tolerance and in the generation of B lymphocytes secreting IgA (Brandtzaeg et al. 1998).

The early colonization of the digestive tract by the microbiota is essential for the postnatal maturation of gut functions and associated immune system. The bacterial colonization depends on maternal factors, especially maternal nutrition and gut microbiota composition and colostrum and milk composition, as well as environmental factors (Campeotto et al. 2007). During the lactation period, the composition of the gut microbiota is unstable and relatively poor, and its diversity is low. The transition to solid food at weaning is an important factor in the diversification of gut microbiota. Thereby, the progressive colonization of the digestive tract by the microbiota has a key role for the development of the piglet and its capacity to respond to infections, ensuring growth and survival of piglets (Chowdhury et al. 2007).

In the current context where the use of antibiotics in pig farming must be limited and represent a major challenge for the sector, nutritional alternatives have demonstrated their effectiveness, and this is particularly the case for the use of prebiotics. Prebiotics are defined as substrates selectively utilized by host microorganisms conferring a health benefit to the host (Gibson et al. 2017). Among these recognized prebiotics, short-chain fructo-oligosaccharides (scFOS) showed interesting beneficial effects on the growth performances and the functionality of the immune system of suckling or weaned piglets consuming them (Le Bourgot et al. 2016; Zhao et al. 2019; Ayuso et al. 2020), likewise when these are administered in the sow’s diet during perinatal life (Le Bourgot et al. 2014; Apper et al. 2016; Le Bourgot et al. 2017; Le Bourgot et al. 2019). An increase in the Ig content of colostrum in sows having received scFOS in gestation was also observed (Le Bourgot et al. 2014; Apper et al. 2016; Le Bourgot et al. 2017; Le Bourgot et al. 2019), suggesting an improved immune transfer favorable for piglets’ health. However, few studies have investigated the effects of these prebiotic fibers on parameters related to the mother’s metabolism, including immune parameters, insulin regulation, lipid metabolism, and her intestinal microbiota. Prebiotics may positively influence sow health and microbiota, enhance colostrum quality, and promote early colonization by beneficial bacteria in the piglets’ gastrointestinal tract. Early microbial colonization is essential for piglet growth and survival during critical developmental stages.

In order to better understand the effect of scFOS distributed during the peripartum period, sows were supplemented with scFOS during the end of gestation and throughout lactation. The impact of such supplementation on sow performance, milk production, and gut microbiota composition was evaluated early in life as well as subsequent consequences on health and growth parameters of piglets.

## Material and methods

### Animals and housing

The study was conducted in accordance with the ethical standards and recommendations for accommodation and care of laboratory animals covered by the European Directive 2010/63/EU on the protection of animals used for scientific purposes and the Belgian royal decree KB29.05.13 on the use of animals for experimental studies. This experiment included 30 sows (Danbred hybrid) with a parity between 1 and 6 and their litters (Piétrain × Danbred hybrid) (farm Degroote; Bissegem, Belgium).

Piglets were ear tagged at birth for individual tracing. All piglets received 1 mL iron dextran (Pharmacosmos A/S, Holbaek, Denmark), 2 mL *Mycoplasma h*. vaccine (Elanco GmbH, Cuxhaven, Germany) and 1 mL Porcine Circovirus vaccine (Boehringer Ingelheim Vetmedica GmbH, Ingelheim, Germany) at three days of age by intramuscular route. At the same time, piglets were orally administered 1 mL of an anticoccidial (Bayer SA-NV, Diegem, Belgium), and their tails were docked. Males were not castrated. During lactation, piglets were offered a dry commercial creep feed (AVEVE Veevoeding), providing 15 MJ of metabolizable energy ME per kg of feed, from d7 post-partum until weaning at d23 post-partum. At weaning, the eight median piglets per litter (determined by individual weighing) were transferred to the weaning facilities and housed 1 L per pen, giving ten pen replicates per treatment.

### Experimental diets

All sows received a standard commercial feeding program (AVEVE Veevoeding; Merksem, Belgium), providing 13.8 MJ of ME per kg of feed. The program consisted of a composed of a gestation diet (3 kg per day in one meal) until d7 pre-partum, a transition diet (3 kg per day in two meals) from d7 pre-partum until d2 post-partum, and a lactation diet (3 to 7.5 kg per day in two to five meals) from d2 post-partum until weaning at d23 post-partum. Sows were allocated to one of three dietary treatments based on their parity, body weight (BW), and back fat (BF) thickness in order to stratify for these parameters and reach a sample size of 10 sows per treatment. Sows were supplemented with 0.15% scFOS (>95% scFOS; Beghin-Meiji, France) from d28 pre-partum to d2 post-partum (gestation and transition diet), where after the dose of scFOS was reduced to 0.10% during further lactation (lactation diet) (low scFOS). Next, scFOS was supplemented at 0.33% from d28 pre-partum until d2 postpartum and 0.15% during further lactation (mid scFOS). The scFOS were a combination of 1-kestose (GF2) (about 37%), nystose (GF3) (about 53%), and 1F-β-fructofuranosyl nystose (GF4) (about 10%). In the control treatment (CTRL), sows received maltodextrin as placebo (0.15% from d28 pre-partum until d2 post-partum and 0.10% during further lactation). Supplementation was performed by means of top-dressing to each meal. All diets were dried pelleted diets.

All piglets were fed the same commercial weaner diet and starter diet (AVEVE Veevoeding). Diets were cereal and soy-based meals with additional high-quality protein and lactose sources, containing no in-feed antibiotics or pharmacological doses of zinc oxide. The weaner and starter diets were offered from d0–14 and d14–42 post-weaning, respectively.

### Measurements and sample collection for sows

At d36 pre-partum, sows were weighed, and BF thickness was measured using a pulsing ultrasonic apparatus (Renco Lean Meter; S.E.C. Repro Inc., Québec, Canada). Measurements were performed by the same person on standing sows at the P2-position after hair removal. Sow’s BW and BF were also measured at d7 pre-partum, d2 post-partum (only BW), and at weaning. Sows were subjected to a daily fecal consistency and constipation score from d7 pre-partum until weaning. Sows were categorized as either 0: no feces, 1: watery to soft, unformed stool that assumes shape of container; 2: soft, formed, moist feces to dry feces that retain shape; and 3: hard and dry feces, pellets. If sows exhibited more than 3 consecutive days of score 0 (no feces) around parturition, it is considered severe constipation. During farrowing, the rank, time of birth and BW (BiW) of each piglet born alive and stillborn was recorded, as also the total farrowing duration, number of piglets born and born alive and stillborn per litter. No farrowing induction was performed.

Average daily feed intake (ADFI) for sow was calculated individually for the gestation (d28 pre-partum until parturition) and lactation (parturition until weaning) period.

Sows were sampled for colostrum (30 mL) at the onset of partus. Colostrum was collected from all teats on one side of the udder and without the use of oxytocin. Milk samples were collected at 48 h and weaning in a similar way but with the intramuscular administration of oxytocin (2 mL, 10 IU/mL). Colostrum and milk samples were stored at −20 °C until further analysis for their nutritional composition and immune quality. Secondly, sow blood samples were taken after overnight fasting the morning following 48 h and at weaning. Blood samples were drawn using 30 mL syringes and 20 G × 1.5” needles at the jugular vein and were transferred into 4 mL EDTA, NaF and serum vacutainers. Plasma was immediately harvested from EDTA and NaF vacutainers after centrifuging (3,000 × *g*, 15 min) and was stored at −20 °C. Serum vacutainers were allowed to clot during 5 h at room temperature, whereafter samples were centrifuged (3000 × *g*, 15 min) and serum was stored at −20 °C. EDTA plasma was analyzed for insulin, leptin, non-esterified fatty acids (NEFA), and triglycerides. Glucose was determined in NaF plasma. Serum was used for the analysis of IgA and IgG. Next, fresh fecal samples were collected from the rectum at 48 h. Subsamples were immediately stored in 15 mL and 2 mL recipients, frozen in liquid nitrogen, and stored at −80 °C. One subsample at d2 postpartum was acidified to pH 2 with 2% (w/w) of 6 molar H_2_SO_4_ to stop fermentation pending analysis for bacterial metabolites. Other subsamples were used for microbiome analysis.

### Measurements and sample collection for piglets

Piglets were weighed individually at 24 h and 48 h after the onset of partus. Piglets’ rectal temperature was measured at 24 h, whereas this was measured in sows at the onset of partus and 12-18 h after the onset of partus. Litter size was standardized to 13 piglets at 48 h by cross-fostering within treatment group or removal of piglets: the smallest supernumerary piglets were removed from the litter, and mean litter body weight was stratified between the treatments to obtain a similar mean litter weight between the treatments (1570 ± 90, 1560 ± 80, 1590 ± 80 g for treatments CTRL, Low scFOS, and Mid scFOS, respectively). The piglet fecal consistency score was visually assessed from birth until weaning on litter level according to the following scoring system: 1 = hard or slightly moist feces, clearly formed, normal; 2 = moist or soft feces, but still with a definite form, sticky; and 3 = watery or liquid feces, unformed, diarrhea. If feces of different consistency in a pen were observed, the highest score present was retained as data.

Creep feed intake was determined by weighing the amount of feed offered daily and by collecting and weighing the feed remaining in the feeder at the end of the trial. Feed wastage was minimized by manually providing small portions several times per day to ensure constant availability of fresh feed while limiting losses.

After weaning, piglets were weighed individually at d14 and d42 post-weaning. Concurrently, feed intake was registered at the pen level by weighing offered and residual feed. Animals were inspected daily by the same trained individual for general health and a fecal consistency score according to the ordinal piglet scoring system described above. Antibiotic treatments were limited to individual intramuscular injections and were registered for sows and piglets during the entire experiment.

Average daily gain (ADG) of piglets was calculated between birth, 24 h post-partum, 48 h post-partum, weaning, d14 and d42 post-weaning. Between d2 and weaning (d23), the ADG was calculated using the weight of the piglets selected after the litter standardization. The piglet ADFI and feed conversion ratio (FCR) were calculated for following intervals; d0-d14, d14–42, and d0–42 post-weaning. Survival rate was evaluated between birth, 48 h, weaning, d14 and d42 post-weaning.

Blood was also collected from two median piglets per litter at 48 h by puncture of the jugular vein, using 4 mL serum vacutainer tubes and a 22 G × 1.5” needles. Serum was collected after 5 h clotting at room temperature and centrifuging (3,000 × *g*, 15 min). Samples were stored at −20°C until the analysis of IgA and IgG levels. At the same time, two 1 g aliquots of feces were collected from the same animal by rectal stimulation with a cotton swab. One subsample was acidified as described above pending the analysis for bacterial metabolites. The second subsample was subjected to microbiome analysis.

### Estimated measurements

Sow’s lactation efficiency was calculated according to (Bergsma et al. 2009) but for the interval 48 h postpartum (after weighing sows and standardization of litters) until weaning. Lactation efficiency was calculated as the ratio between the output in litter growth and the energy input of sows expressed in MJ metabolizable energy per day. The energy input was calculated as the total energy intake from feed plus the energy contributed from maternal tissue mobilization minus energy required for sow maintenance. Energy from tissue mobilization was calculated using the sow’s BW at 48 h and weaning and the BF at d7 pre-partum and weaning. Sow’s BW at 48 h and weaning were corrected for water content in the mammary gland on the basis of previous experiments (Kim et al. 1999a; Kim et al. 1999b; Kim et al. 2000) equations derived by (Bergsma et al. 2009). Maintenance energy requirement of the sow was derived from its metabolic BW (Noblet et al. 1990). Energy output was based on WG from 48 h to weaning for dead and live piglets and energy requirements for piglets during that period (Everts and Dekker 1994a; Everts and Dekker 1994b) subtracted with the amount of energy provided by creep feed. Finally, the milk energy balance, defined as the difference between milk energy output and energy required for gain and maintenance of piglets, was calculated following the equations of (Bergsma et al. 2009) and efficiency rates of (Noblet et al. 1990) and (Mullan et al. 1989).

Estimated measures of colostrum intake (CI) per piglet were calculated using a formula designed by Theil et al. (2014): CI = –106 + (2.26 × WG) + (200 × BiW) + (0.111 × TS) – (1,414 × WG)/TS + (0.0182 × WG)/BiW. Time suckling (TS) was calculated from the exact time of birth until 24 h after the birth of the first piglet. Weight gain (WG) was the difference between BiW and BW at 24 h. CI and WG are expressed in grams, BiW in kilograms and TS in minutes. Colostrum production of the sow is considered the sum of the individual colostrum intakes of each piglet within the litter (Theil et al. 2014).

### Laboratory analysis

#### Diets

Dry matter content in diets was determined by oven drying at 103 °C until constant weight (ISO 6496:1999) (Table 1). Total nitrogen (N) content was determined by the Kjeldahl method (ISO 5983-1:2005). Crude protein content was calculated by multiplying total N with 6.25. Ash was analyzed by incineration at 550 °C for 4 h in a combustion oven (ISO 5984:2002). Ether extract, a measure for crude fat, was analyzed gravimetrically after extraction with diethyl ether with a Soxhlet system (ISO 6492:1999). Crude fiber content was determined by using the method with intermediate filtration (ISO 6865:2000).

**Table 1:** Proximate analysis of basal diets of sows (given in % as fed).

	Gestation diet	Transition diet	Lactation diet
**Dry matter**	88.5	89.9	89.2
**Crude protein**	12.9	13.5	16.2
**Ash**	5.3	5.8	5.7
**Ether extract**	4.4	3.9	4.7
**Crude fiber**	8.6	12.1	8.1

#### Nutritional composition of colostrum and milk samples

Total nitrogen (N) content was determined by the Kjeldahl method. Crude protein content was calculated by multiplying total N with 6.25. Ether extract (EE), a measure for fat content, was analyzed gravimetrically after extraction with diethyl ether with a Soxhlet system. Lactose content was determined colorimetrically using the phenol-sulfuric acid reaction.

#### Colostral and milk TGF-β1 assay

The cytokine TGF-β1 was determined in colostrum and milk by using the Pig Porcine TGF Beta 1 ELISA Kit PicoKine (EK0513-PO; Boster; Pleasontan, CA, USA).

#### Immunoglobulin assay

Serum samples from sows and piglets as well as samples of sow colostrum and milk were evaluated for IgG and IgA levels. Concentrations of Ig were quantified using swine IgG or IgA ELISA Quantification Kit (BET E101-104 and BET E101-102, respectively) (Bethyl Laboratories; Montgomery, TX, USA). Samples were all diluted in tris buffered saline 1% bovine serum albumin 0.05% Tween-20.

#### Insulin, leptin, NEFA, triglycerides, and glucose analysis

Insulin (LS-F3456) and leptin (LS-F22387) concentrations in serum of sows were measured by using ELISA kits (Lifespan BioSciences; Seattle, WA, USA). Biochemical measurements (NEFA, triglycerides and glucose) were assessed using routine methods at the diagnostic laboratory Animal Health Care (DGZ; Torhout, Belgium). HOMA-IR was calculated with the following formula:


$$\mathrm{HOMA}-IR=\;\frac{\mathrm{Insulin}\;(\mu IU/mL)\times \mathrm{Glucose}\;(\mathrm{mmol}/L)}{22.5}$$

#### Microbiome analysis by 16S-Illumina sequencing

The microbiota profiling was established by 16S-targeted Illumina sequencing analysis. The 16S rRNA gene V3-V4 hypervariable regions were amplified by PCR using primers 341F (5′-CCT ACG GGN GGC WGC AG-3′) and 785Rmod (5′-GAC TAC HVG GGT ATC TAA KCC-3′), adapted from Klindworth et al. (2013). The original genomic DNA extracts were diluted in DNase/RNase/protease-free water to obtain a concentration of 50 ng/μL and 30 μL was sent out to LGC genomics GmbH (Berlin, Germany) for library preparation and sequencing on an Illumina Miseq platform with v3 chemistry with the primers mentioned above. The 16S rRNA gene sequences in this study were deposited in the NCBI Sequence Read Archive (SRA) database with the accession number SUB1052053. Originally, 3518872 raw reads were obtained. The raw reads were submitted to the DADA2 package (version 1.20.0) (Callahan et al. 2015) in R (version 3.3.1, http://www.r-project.org). The Divisive Amplicon Denoising Algorithm (DADA) is based on the identification of single nucleotide sequence variants and provides higher resolution (Callahan et al. 2017). The raw sequences were quality trimmed and filtered, error models were constructed, amplicon sequence variants (ASV’s) were inferred, and forward and reverse reads were merged, and chimeras were removed following default settings or adjusted. It resulted in 2,078,685 reads (5,026–65,744 per sample). The SILVA (release 138.1; https://www.arb-silva.de/documentation/release-1381/) (Quast et al. 2012) was used for taxonomy assignment. Taxonomy data and metadata were merged into a phyloseq object applying the Phyloseq package (version 1.36.0) (McMurdie and Holmes 2013) in R. Low count ASV’s were removed with a threshold of 0.01% and the ASV table was normalized to 4497 reads by single rarefaction (Figure S1). A total of 625 ASV’s, condensed into 10 phyla, 54 families, 148 genera, and 219 species, were used in the downstream analysis. PCoA plots based on Bray-Curtis distance were employed to visualize differences in bacterial community composition between different groups at ASV and genus level (beta diversity). The diversity within bacterial communities per group (alpha diversity) was assessed with the Chao1 index (richness), Shannon index (evenness), and reciprocal Simpson index (diversity) at ASV and genus levels in Phyloseq. Flow cytometry analysis was conducted on all fecal samples to determine the number of bacterial cells following making a 10-fold dilution series in anerobic phosphate-buffered saline and staining with SYTO 24 (van den Abbeele et al. 2020) (Figure S2). These numbers were used to convert relative abundances into quantitative measurements of bacterial presence. However, as no significant differences in number of bacterial cells between treatments within animal category was found, and as subsequent statistical analysis of relative abundances or absolute numbers revealed very similar significances, it was chosen to continue working with relative abundances only.

#### Analysis of short-chain fatty acids (SCFA) in fecal samples

SCFA analysis of fecal samples was performed on GC after extraction with 10% formic acid with ethyl butyric acid as the internal standard, as described by (Castro-Montoya et al. 2012).

### Statistics

Statistical analysis was performed using GLM mixed procedures, including treatment (CTRL, Low scFOS, Mid scFOS) and parity class as fixed factors. Additionally, covariates were introduced in analyzing performance data prior to weaning, if significant. Two covariates were used, depending on the parameter, namely N° piglets born alive and lactation length for data before and after standardization of litters (at d2), respectively. Least square means were separated by contrasts (Tukey test). Statistical significance was defined as *P* < 0.05, and trend was reported as *P* ≤ 0.10, with the *P*-value corresponding to the linear effect.

For the microbial composition following 16S rRNA amplicon sequencing, statistical analyses were performed in R using the packages Phyloseq and vegan for community analysis (version 2.5.7) (Dixon 2003). Significant differences in bacterial community composition between different groups at ASV and genus level (beta diversity) were identified with pairwise permutational PERMANOVA on Bray-Curtis distance with Bonferroni correction, using the adonis2 function (vegan). ANOSIM (vegan) procedures were employed to test for differences in community composition among sows and piglets within treatment. ANOSIM is a permutation-based test where the null hypothesis states that within-group distances are not significantly smaller than between-group distances. The test statistic (R) can range from 1 to 0, with a value of 1 indicating that all samples within groups are more similar to each other than to any other samples from different groups. R is ≈ 0 when the null hypothesis is true, that distances within and between groups are the same on average. Statistical differences in relative bacterial abundances at phylum, family, genus, and species levels between treatments were tested by non-parametric Kruskal–Wallis tests, multiplicity was corrected using the Benjamini-Hochberg false discovery rate (FDR, with FDR = 0.05 for 16S rRNA gene profiling) (Lee and Lee 2018).

## Results

### Performances of sows and litter parameters and growth of progeny during lactation and post-weaning

**Figure 1:**
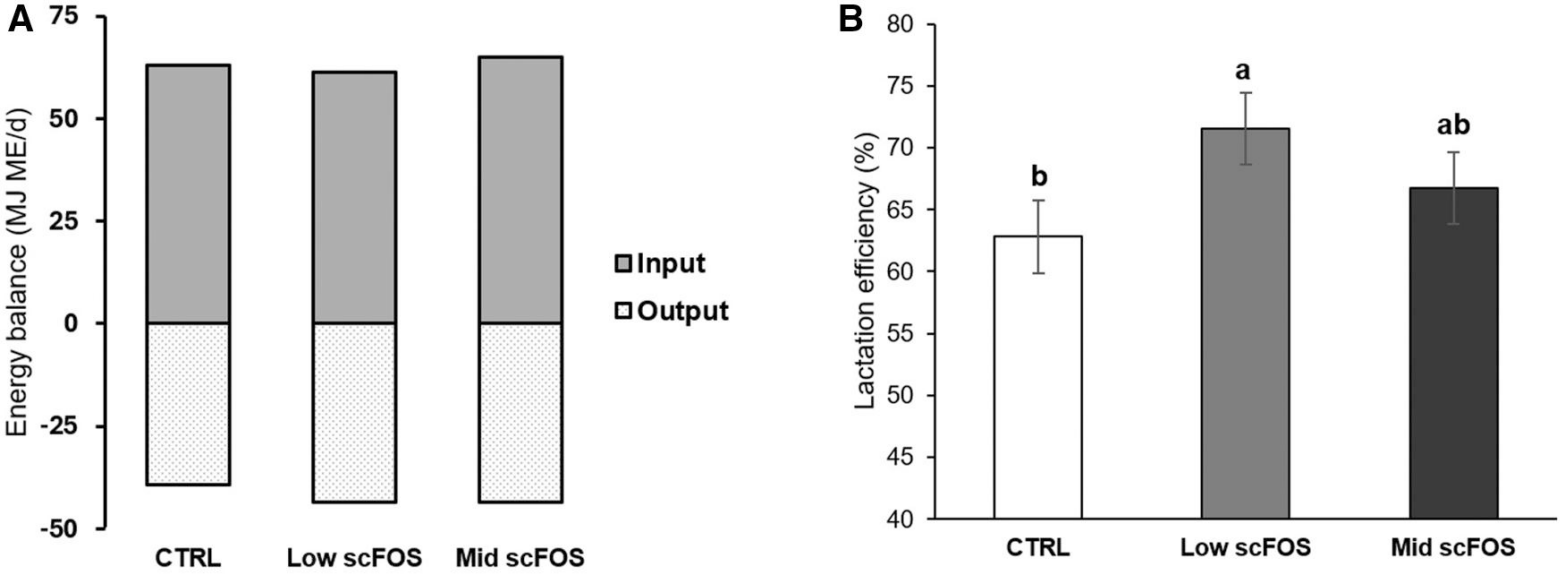
Effect of treatment of lactation efficiency in sows fed the experimental diets between 48 h post-partum and weaning.

No difference was observed between the three groups of sows on BW, BF thickness, fecal score, feed intake, and the farrowing duration (Table 2). Litter parameters such as the total number of piglets per litter, number of stillborn and born alive were not impacted by maternal scFOS supplementation. Although no statistically significant effect of maternal scFOS supplementation on piglet BW during lactation was detected (Table 3), numerically differences at birth (+112 g; +9.2%) and at weaning (+440 g; +7.3%) were observed in the mid scFOS group compared with CTRL. A tendency for a reduction of mortality rate between D2 and D23 was observed with Mid scFOS supplementation leading to a trend increase in the number of weaned piglets (both *P* = 0.085). According to model‑based estimates, using the calculation method previously described, supplementation of the sow with the lowest dose of scFOS significantly increased the lactation efficiency (+13%; *P *< 0.05; Figure 1), resulting in superior output, while Mid scFOS was intermediate.

**Table 2:** Effect of treatment on performances of sows and litter parameters (*n* = 10/group).

Item	CTRL		Low scFOS		Mid scFOS		*P*-value
	Mean	SE	Mean	SE	Mean	SE	
**Sow performances**							
**Parity**	2.9	0.3	3.1	0.4	3.0	0.3	0.972
**BW D28, kg**	234	6	238	5	239	5	0.504
**BW D7, kg**	277	9	270	9	265	9	0.362
**BW D2, kg**	240	5	239	4	241	4	0.802
**BW D23, kg**	222	5	223	5	223	5	0.902
**Backfat D28, mm**	12.2	0.9	13.1	0.8	12.9	0.8	0.576
**Backfat D7, mm**	13.6	1.0	13.2	0.9	13.1	0.9	0.706
**Backfat D23, mm**	10.6	0.7	11.0	0.7	10.0	0.7	0.617
**Sow daily fecal score**	2.7	0.1	2.6	0.1	2.6	0.1	0.154
**ADFI during gestation, g/d**	3000	0	3000	0	3000	0	—
**ADFI during lactation, g/d**	5190	90	5170	90	5200	90	0954
**Duration of farrowing, min**	201	31	224	30	240	30	0.382
**Litter parameters**							
**Number of piglets born alive**	17.0	1.5	19.3	1.4	19.7	1.4	0.201
**Number of stillborn piglets**	0.9	0.3	0.5	0.3	0.9	0.3	1.000
**Total colostrum intake, g**	7760	480	7050	450	7640	450	0.855

BW, body weight; D, Day with partus as reference day 0; CTRL, control group; Low scFOS: sows supplemented with 0.15% scFOS from d28 pre-partum to d2 post-partum (gestation and transition diet) and 0.10% during lactation (lactation diet); Mid scFOS: sows supplemented with 0.33% scFOS from d28 pre-partum to d2 post-partum (gestation and transition diet) and 0.15% during lactation (lactation diet); ADFI, average daily feed intake (g/d).

**Table 3:** Effect of treatment on performances of piglets in lactation (*n* = 10).

Item	CTRL		Low scFOS		Mid scFOS		*P*-value
	Mean	SE	Mean	SE	Mean	SE	
**Number of piglets at D2**	16.0	0.6	17.0	0.5	16.7	0.5	0.451
**Number of deaths between D0 and D2**	3.0	0.6	1.8	0.5	2.3	0.5	0.387
**Number of piglets weaned**	12.3	0.2	12.4	0.2	12.9	0.2	0.085
**Number of deaths between D2 and D23**	0.6	0.2	0.6	0.2	0.2	0.2	0.085
**BW D0 for all piglets alive, g**	1209	62	1264	58	1321	62	0.218
**BW D1 for all piglets alive, g**	1285	63	1343	58	1389	59	0.250
**BW D2 for all piglets alive, g**	1417	62	1488	58	1538	58	0.172
**BW D23 for standardized litter, g**	6050	300	6260	280	6490	290	0.307
**Daily growth from D0 to D2, g/d**	91	12	87	11	98	11	0.655
**Daily growth from D2 to D23, g/d**	206	11	214	10	221	11	0.325
**Colostrum intake, g**	462	33	424	31	467	31	0.918
**Creep feed intake, g**	366	50	306	47	334	47	0.650
**Rectal temperature D1, °C**	38.3^a,b^	0.1	38.2^b^	0.1	38.6^a^	0.1	0.171

BW, body weight; CTRL, control group; D, day with partus as reference day 0; Low scFOS, group supplemented with the lowest dose of scFOS; Mid scFOS, group supplemented with the highest dose of scFOS. Treatments without common letter are significantly different at *P* < 0.05.

**Figure 2:**
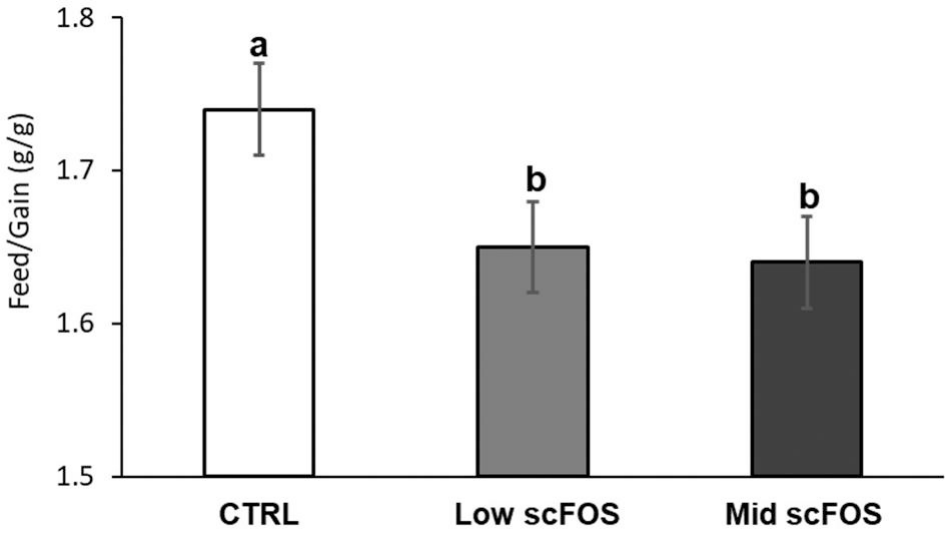
Effect of treatment on feed conversion ratio during 14–42 d post-weaning period of piglets originating of sows fed the experimental diets.

A significant reduction of FCR in both groups of weaned piglets whose mothers were supplemented with scFOS between D14-42 post-weaning was established (*P *< 0.05; Figure 2), while other post-weaning performance indices were not significantly affected, nor number of antibiotic treatments (Table S1).

### Nutritional and immune quality of colostrum and milk of sows

**Figure 3:**
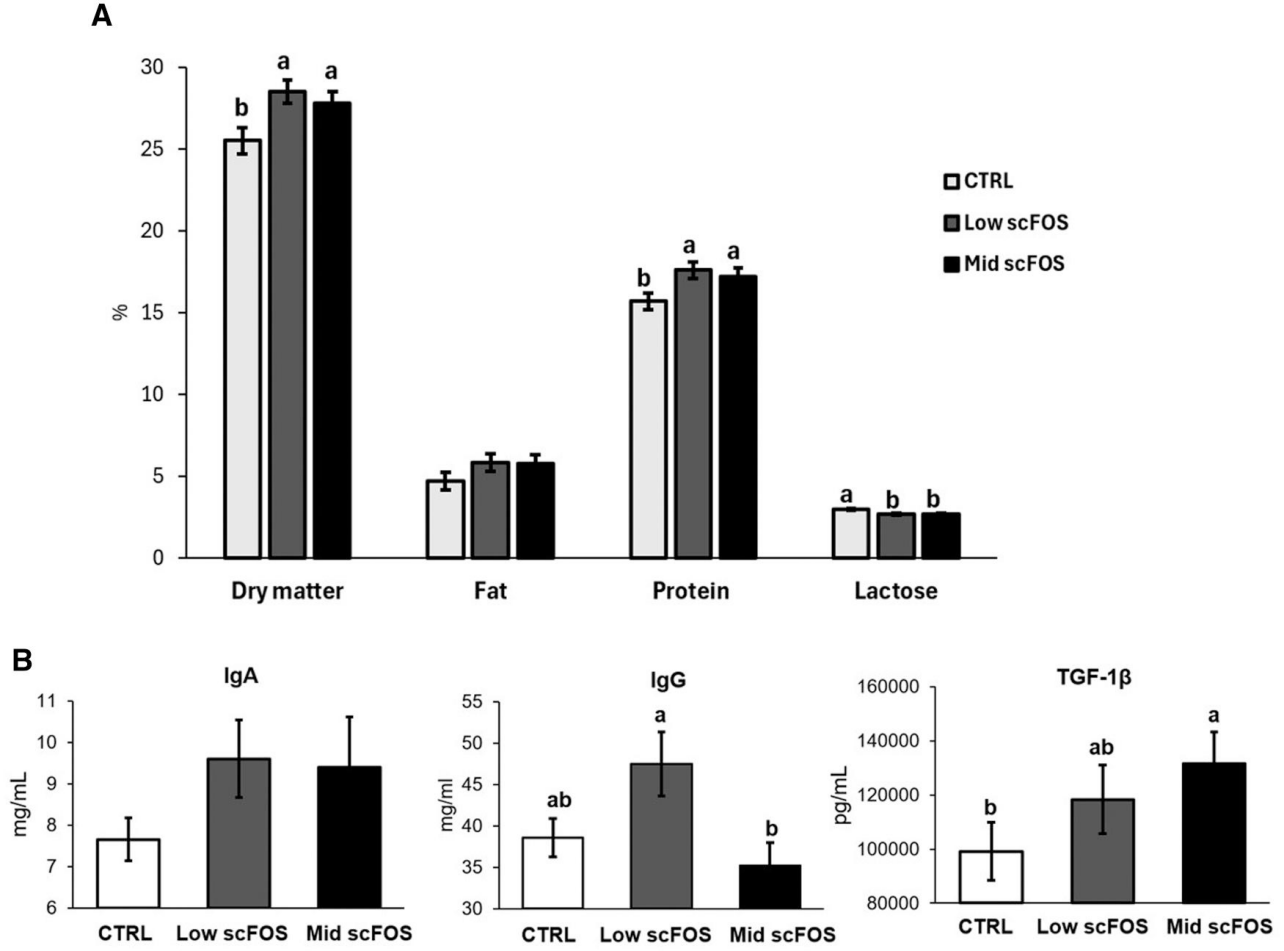
Effect of treatment on nutritional composition (A) and immune quality (B) of colostrum of sows.

The intake of scFOS in sow diet increased the dry matter and protein content in colostrum while it reduced its lactose level (low scFOS and mid scFOS vs CTRL; *P *< 0.05), without modification of lipid content (Figure 3A). No modification in major nutrients has been observed in milk collected at D2 and D23. IgG concentration was increased in low scFOS group (+17%) while TGF-β1 concentration was increased in Mid scFOS group (+33%; *P *< 0.05) as compared to CTRL, without alterations of IgA content (Figure 3B). In the milk at D2, IgG concentration was much lower than in colostrum (CTRL: 40.2 g/L in colostrum vs 2.6 g/L in milk at D2). A reduction of this concentration was observed in Low scFOS group as compared to CTRL (CTRL: 2.6 g/L, Low scFOS: 1.8 g/L; *P *< 0.05), while IgA and TGF-β1 concentrations were not different between groups. Supplementation with scFOS did not impact the milk immune parameters at D23 (data not shown).

### Metabolism and immunity of sows at d 2

The intake of scFOS in the diet of sows tended to reduce triglycerides concentration at D2 in both supplemented groups in comparison to CTRL group (*P *= 0.058; Table 4). We also observed a numerical reduction of HOMA-IR index in sows supplemented with the lowest dose of scFOS due to a numerical decrease in plasma insulin concentration at D2, but the effects were not significant. No differences were observed concerning IgA and IgG concentrations in serum of sows at D2, neither in serum of piglets at D2. Supplementation with scFOS did not impact metabolism and immunity of sows at d 23 (data not shown).

**Table 4:** Effect of treatment on metabolism and immune parameters in the blood of sows at d 2 (*n* = 10).

Item	CTRL		Low scFOS		Mid scFOS		*P*-value
	Mean	SE	Mean	SE	Mean	SE	
**Insulin, μIU/mL**	13.2	2.7	8.1	2.5	14.8	2.7	0.694
**Glucose, mmol/L**	4.21	0.17	4.10	0.16	4.02	0.16	0.412
**HOMA-IR**	2.6	0.6	1.5	0.5	2.7	0.6	0.846
**Leptin, ng/mL**	1.30	0.28	0.83	0.27	1.22	0.28	0.854
**NEFA, mmol/L**	0.59	0.09	0.56	0.09	0.75	0.09	0.237
**Triglycerides, mg/dL**	31.8	1.7	25.7	1.6	26.9	1.7	0.058
**IgA, mg/mL**	1.50	0.18	1.57	0.18	1.42	0.17	0.754
**IgG, mg/mL**	14.1	1.6	17.7	1.5	15.4	1.5	0.562

CTRL: control group; Low scFOS: group supplemented with the lowest dose of scFOS; Mid scFOS: group supplemented with the highest dose of scFOS; HOMA-IR: homeostasis model assessment of insulin resistance; NEFA: non-esterified fatty acids.

### Gut microbiota composition and fermentative activity of sows and piglets at d 2

**Figure 4:**
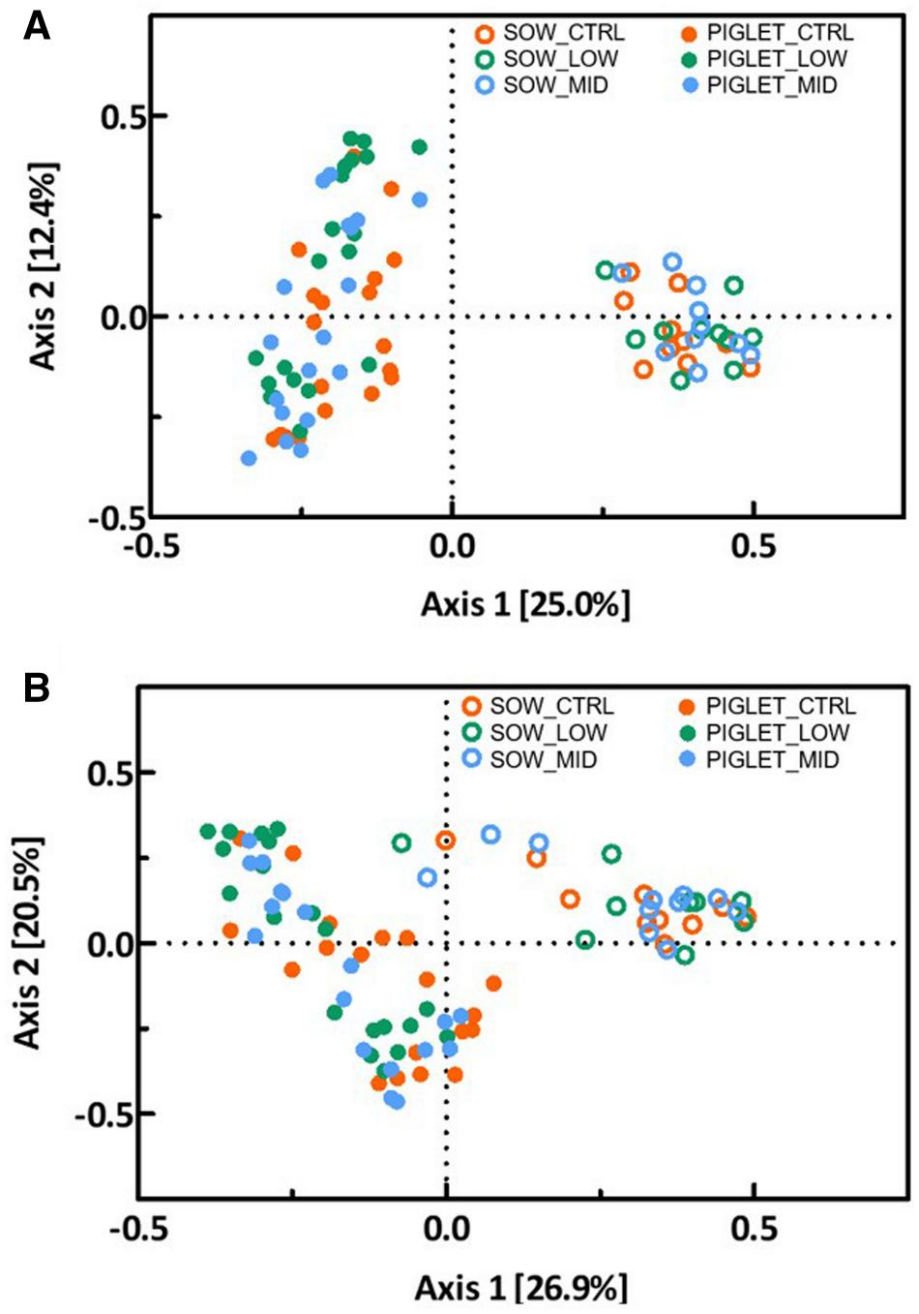
Effect of treatment on bacterial community composition in feces in sows 2 d post-partum and in feces in piglets at d 2 after birth at Amplicon Sequence Variant (ASV).

**Figure 5:**
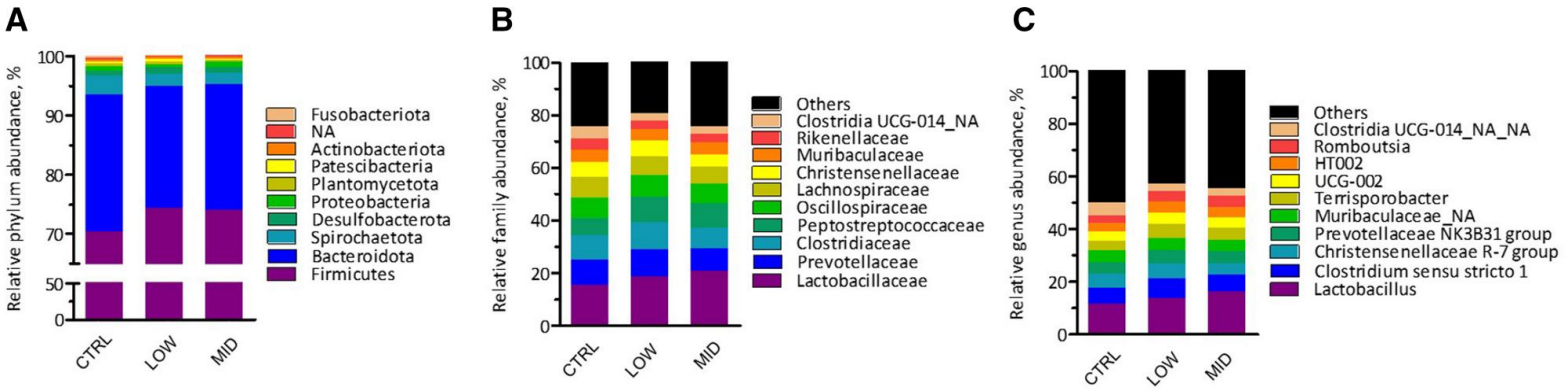
Effect of treatment on relative abundance (%) of bacterial taxa (a, phylum; b, family; and c, genus level) in feces in sows 2 d post-partum.

**Figure 6:**
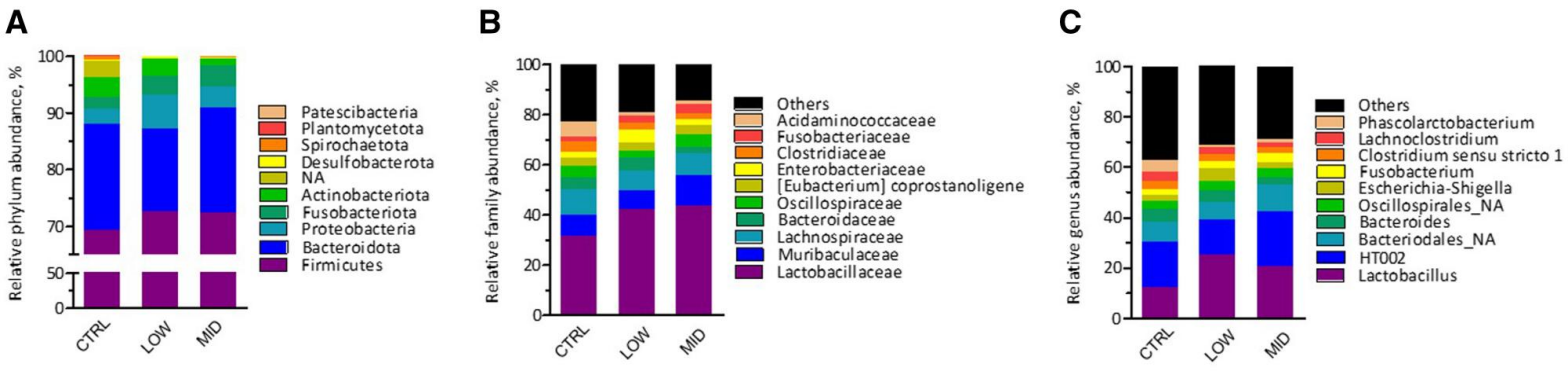
Effect of treatment on relative abundance (%) of bacterial taxa (a, phylum; b, family; and c, genus level) in feces in piglets at d 2 after birth.

The bacterial community composition in feces of sows at D2 was markedly different from that of their progeny at the same day, both at ASV level and genus level (*P *< 0.05; Figure 4), yet within the animal category, no separation of community composition could be observed. Most segregation of subjects was obtained along Axis1, representing 25.0% and 26.9% of variance at ASV and genus level, respectively. Furthermore, supplementation of scFOS did not affect indices of alpha diversity nor in sows or piglets (Figure S3). Most abundant taxa in feces of sows are presented in Figure 5 and in feces of piglets in Figure 6, while statistical inferences in relative abundances between treatments are given in Tables S2 and S3. The phyla Firmicutes (with families Lactobacillaceae, Clostridiaceae, Oscillospiraceae, and Peptostreptococcaceae) and Bacteriodota (with families Prevotellaceae, Muribaculaceae, and Rikenellaceae) dominated in feces of sows. However, only one taxon, i.e., the genus *Sphaerochaeta* (not assigned species), was significantly altered by treatment in feces of sows. It represented 0.020% of reads in Low scFOS, while in CTRL and mid scFOS this was 0.156% and 0.207%, respectively (*P *< 0.05) (Table S2).

Continuing into the fecal microbiome of the piglets, numerous differences were seen (Table S3). Again, Firmicutes and Bacteriodota were the main phyla, yet other phyla such as Proteobacteria, Fusobacteriota, and Actinobacteriota were present in higher abundances as compared to sows. Within the Spirochaetota, the genus *Treponema* was significantly reduced by the lowest dose of scFOS (*P *< 0.05), with Mid scFOS being intermediate, while the genus *Sphaerochaeta* was here only numerically lower but at both scFOS levels. Dose-dependent effects were found on members of the Actinobacteriota, it refers to families Actinomycetaceae, represented by the genus *Actinomyces*, and Eggerthellaceae. The latter showed an abundance of 1.16%, 0.87%, and 0.36% for CTRL, low scFOS, and mid scFOS, respectively. *Bacteroides uniformes* and *Rikenellaceae RC9 gut group NA* (Bacteriodota) were also reduced by scFOS intake. Various changes within the Firmicutes were observed. Most prominent were the increases in the genera *Lactobacillus*, caused by changes in abundance of *Lactobacillus delbrueckii* and *Lactobacillus johnsonii*, *Ligilactobacillus*, and *Limosilactobacillus*; yet in some cases with clear dose effect and in other cases with low scFOS being highest. Furthermore, the genus *Peptostreptococcus* was increased by mid scFOS, whereas the genus *Phascolarctobacterium* was dramatically decreased by both scFOS doses in sow’s diet. Finally, *Pasteurella aerogenes* (Proteobacteria) was elevated by supplementing scFOS, its abundance was 0.08%, 0.36%, and 0.75% for CTRL, low scFOS, and mid scFOS, respectively. Analysis of similarities between sows and their progeny within treatment resulted in higher statistic R values for scFOS groups as compared to CTRL (Table 5). An R value between 0.75 and 1.00 may be considered as bacterial communities that are highly different, while 0.50 < R < 0.75 is different and 0.25 < R < 0.50 is different with some overlap. The results may suggest that supplementing scFOS to sows resulted in larger differences between microbiome of dam and progeny. This is confirmed by the lower number of common ASV’s between dam and progeny in low scFOS and mid scFOS as compared to CTRL.

**Table 5:** Effect of treatment on test for similarities and percentage of common taxa between microbiome composition of sows and piglets within treatment.

Item	CTRL	Low scFOS	Mid scFOS
**ASV level**			
**ANOSIM test**			
**Statistic R**	0.697	0.864	0.875
***P*-value**	<0.001	<0.001	<0.001
**Percentage of ASV’s in common, %**	24.1	15.2	19.2
**Genus level**			
**ANOSIM test**			
**Statistic R**	0.372	0.577	0.598
***P*-value**	<0.001	<0.001	<0.001
**Percentage of genera in common, %**	46.3	35.4	44.0

**Figure 7:**
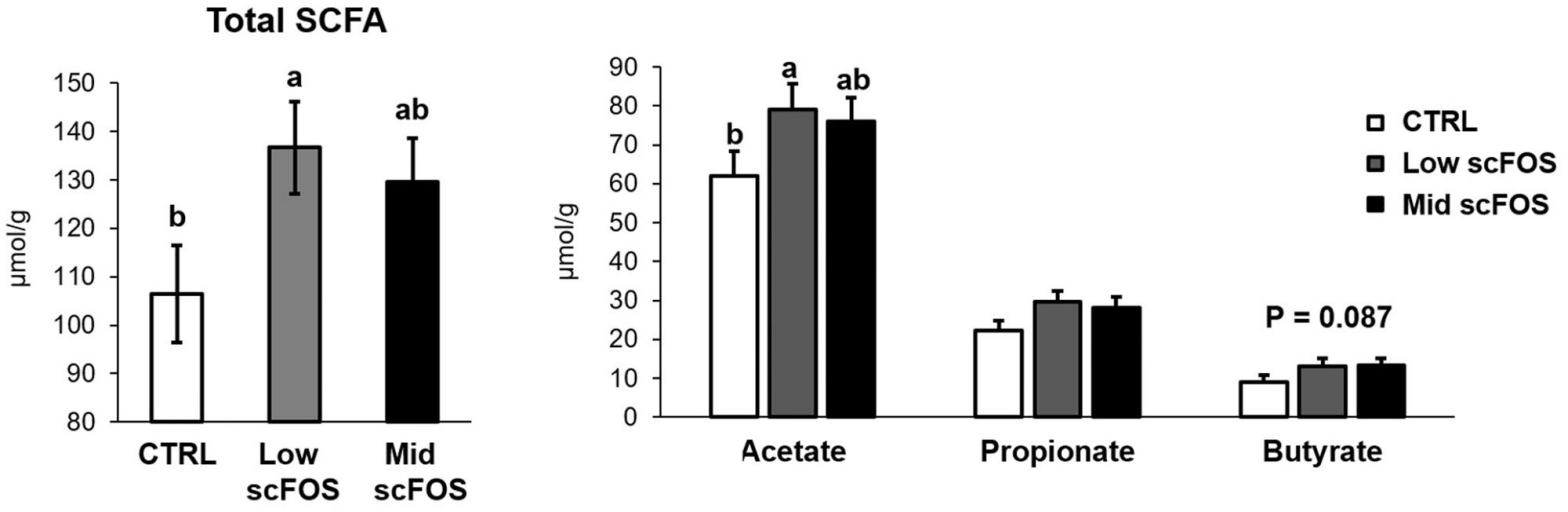
Effect of treatment on short-chain fatty acids (SCFA) concentrations in feces in sows 2 d post-partum.

As opposed to microbiota composition, scFOS supplementation affected largely the fermentative activity of the gut microbiota evaluated by the measure of SCFA in feces (Figure 7). A significant increase in SCFA concentration was observed in low scFOS group of sows at D2 in comparison to CTRL group (*P *< 0.05), mid scFOS group being intermediate. This increase in total SCFA concentration originated from a higher level of acetate in low scFOS group (*P *< 0.05) and a tendency for an increase in butyrate in low scFOS and Mid scFOS groups (*P *= 0.087). Total SCFA in feces of suckling piglets at D2 ranged between 26.4 to 33.0 μmol/g, but without treatment differences, nor were contents of individual SCFA affected by sows’ diet (data not given).

## Discussion

### Colostrum quality

Firstly, this study demonstrated that the consumption of scFOS by gestating sows during the last 4 wk before farrowing improved the nutritional composition of colostrum as well as its immune quality, highlighted by an increase in the protein content, in IgG and in TGF-β1 concentrations. The increase in colostral immune markers, without a marked systemic response, may be related by microbiota-driven modulation of gut mucosal immunity by scFOS, which could enhance local immunoglobulin production subsequently transferred to the mammary gland via the entero-mammary immune link, as previously observed in bitches (Adogony et al. 2007).

TGF-β1 plays a key role in immune regulation by modulating T and B cell responses, while immunoglobulins antibodies are essential for pathogen neutralization (Lodyga and Hinz 2020). This improvement of passive immunity transmitted to newborns confirmed the results previously obtained by other authors (Le Bourgot et al. 2014; Apper et al. 2016; Le Bourgot et al. 2017; Le Bourgot et al. 2019). Interestingly, our study showed that a lower dose of scFOS (0.15% vs 0.33%) was also effective in enhancing the immune quality of colostrum, although the numerical increase in IgA content in our study did not reach significance. The immunity derived from colostrum represents an important component for the development of the piglet, providing it with protection against the pathogens encountered and allowing it to acquire tolerance to food antigens (Salmon et al. 2009). As in a previous study (Le Bourgot et al. 2014), we did not observe changes in milk immune parameters later during the lactation period.

### Piglet microbiota

We then evaluated the early colonization of the intestinal microbiota of the piglet by determining both the composition and the fermentative activity of the microbiota of sows and piglets two days after birth. Our results showed that the composition of the sow’s microbiota was not clearly impacted by scFOS supplementation, although its fermentation activity was increased. In piglets, changes in bacterial composition appeared significantly more marked than in their mothers. Firstly, at the phylum level, maternal scFOS supplementation induced a decrease in Spirochaetota. These bacteria are associated with microbiota imbalance and intestinal inflammation, leading to a higher incidence of diarrhea (Panah et al. 2023; Zhang et al. 2024). At the family level, several bacterial populations that can induce an inflammatory response decreased with scFOS supplementation, such as Spirochaetaceae and Actinomycetes (Panah et al. 2023). A reduction of the controversial family Acidaminococcaceae was also observed: this family is a SCFA-producer (Zhang et al. 2018) but is positively associated with aggressive behavior (Parois et al. 2020), and some of its strains are considered undesirable (Genova et al. 2023). In parallel, the abundance of Eggerthellaceae, which is positively correlated with antioxidant defenses (Feng et al. 2023) and with the release of beneficial bioactive molecules (Rodríguez-Daza et al. 2021), decreased from 1.16% in the control group to 0.36% in mid scFOS.

Nevertheless, the most abundant family, i.e. Lactobacillaceae recorded a numerical increase: from 31.3% to 42.1% and 43.5% for low scFOS and mid scFOS, respectively. The genus *Lactobacillus* increased significantly in low scFOS and numerically in mid scFOS, with a notable increase in the beneficial *Lactobacillus delbrueckii* and *Lactobacillus johnsonii* species. These results revealed a positive effect of such maternal supplementation with scFOS on the early implantation of the microbiota. Indeed, these bacteria from the Lactobacillaceae family are commonly considered beneficial to health. Bacteria like *Lactobacillus* spp. are capable of producing antimicrobial compounds that limit intestinal colonization by pathogens (Reis et al. 2012) and may also increase the expression of mucins that inhibit bacterial uptake of *E. coli*, a well-known major pathogen. The growth of these populations, enhanced by scFOS consumption, could inhibit the growth of other beneficial genera through the production of antimicrobial compounds, competition for substrates, and the environmental changes associated with SCFA production (Pessione 2012; George et al. 2018; Burakova et al. 2022). A previous study showed that the addition of *Lactobacillus* can reduce alpha-diversity and decrease the abundance of the Bacteroidota phylum (Burakova et al. 2022). In our study, a reduction in the Rikenellaceae RC9 gut group, associated with greater growth performance in pigs (Popov et al. 2023), from 1.43% in the control group to 0.21% in Mid scFOS, was observed. As a succinate producer, its reduction could be linked to the reduction of *Phascolarctobacterium*, which is capable of metabolizing succinate into propionate and acetate and avoiding deleterious succinate accumulation (Fernández-Veledo and Vendrell 2019; Popov et al. 2023).

Among the potentially pathogenic genera, *Peptostreptococcus*, found in diarrheic piglets, significantly increased from 0.68% in the control group to 2.01% in Mid scFOS, but some others, such as *Treponema* and *Clostridium sensu stricto 2*, considered diarrhea biomarkers (Han et al. 2019; Zhu et al. 2024), decreased. Globally, microbial changes were characterized by a marked increase in the Lactobacillaceae family, which reached notably high levels (until 43.5%), at the expense of other taxa that were considerably less abundant (generally below 5%), along with a reduction in pathogenic species.

### Microbial activity

In our study, we did not observe changes in SCFA production, probably due to the fact that at 2 d of age, piglets only consumed milk, not enough substrates to produce high amounts of SCFA. However, we can suppose that microbiota changes in early life may maintain until post-weaning period and explain the improvement of gut defenses later in life, once pigs become more mature. Improved gut defenses, related to microbial changes in populations and activity, were previously observed in weaned piglets from sows supplemented with scFOS (Le Bourgot et al. 2017).

Indeed, a previous study showed that maternal supplementation with scFOS during the last month of gestation and during lactation also had an impact on the gut microbiota composition of the 21-day-old piglet and even in the longer term, once the pig was becoming adult. The authors showed an increase in the *Prevotella* genus, not observed in our study (Le Bourgot et al. 2019). This difference may be explained by the fact that *Prevotella* proportion increased later in life, notably after the weaning of pigs, as demonstrated by (Mach et al. 2015). Thus, the age of the piglet (2 d vs 21 d) may be a factor explaining the differential results between studies.

### Early gut microbiota colonization

Overall, these changes in early gut microbiota colonization seem to be beneficial for piglet health as it is oriented toward a profile favorable to the maturation of gut defenses, preventing intestinal barrier disorder, inflammation, and later infection. Interestingly, some similarities in bacterial profiles were observed between sows and their piglets, and this was particularly the case for the *Lactobacillaceae* family. The low scFOS and mid scFOS groups showed numerically higher levels of *Lactobacillaceae* than the sows of the CTRL group, and the same thing was observed in piglets. These microbial profile similarities between sows and piglets suggest that there was a transfer of gut microbiota at birth from sow to piglet, impacting the primary colonization of newborns. It has also been demonstrated in mice supplemented with scFOS, confirming the potential of scFOS administered in the mother to modulate the primo-colonization of neonates (Fujiwara et al. 2008). Nevertheless, in our study, there were few differences between the fecal microbiota of sows in the tested groups, while significant differences were found in their piglets. Constipation that frequently occurs at the end of gestation can affect gut motility (Oliviero et al. 2010), and the fecal microbiota may not be representative of the populations in the gut. In addition, drastic changes in the microbiota can be observed during the days following farrowing, increasing the variability of the samples (Gaukroger et al. 2021). Regarding the microbiota of the neonatal piglets, previous studies have shown that the maternal fecal microbiota accounted for less than 10% of the piglet microbiota at day 1, while the colostrum accounted for 34% (Lim et al. 2023). Changes in colostrum and milk composition could have a significant impact on the microbiota establishment (Le Bourgot et al. 2024).

### Lactation efficiency

It is well-known that nutrition of sows is crucial in preserving reproductive performance. Indeed, voluntary feed intake of highly prolific sows is generally insufficient to cover nutrient requirements for milk production and maintenance of body condition (Boulot et al. 2008). Interestingly, our estimations showed that the supplementation with scFOS increased lactation efficiency of sows corresponding to the percentage of the metabolizable energy through feed intake or mobilization from body stores, above maintenance of the sow, used for piglet growth and piglet maintenance. An association has been established between an improved lactation efficiency and some reproductive characteristics such as a reduction of fat losses, piglets’ mortality, and litter variability at weaning and an increase of piglets’ growth (Bergsma et al. 2009). We also observed slight differences in metabolism of sows supplemented with scFOS, mainly a tendency to reduce HOMA-IR index suggesting a preventive effect of scFOS against insulin-resistance development. These changes in the metabolism of sows resulted in differences in the growth of piglets. A numerical increase in body weight at birth and at weaning was observed in piglets from sows supplemented with scFOS and more pronounced for the Mid scFOS group, whose dose of scFOS was the highest (+ 440 g at weaning).

### Growth performance of the piglets

At the same time, a tendency to reduce the mortality rate during lactation led to a higher number of weaned piglets in the Mid scFOS group. These early changes resulted in a significant improvement in the feed conversion factor in the second phase after weaning in the two groups of piglets born to sows supplemented with scFOS (−5%). Similar results have been obtained previously. In fact, in previous studies, no significant results were obtained on growth of piglets during lactation, but the maternal scFOS supplementation induced a later effect after weaning. An increase in the body weight of weaned pigs was reported at the age of 56 and 77 d following maternal scFOS supplementation during the last month of gestation and all the lactation period (Le Bourgot et al. 2016). The same effect was observed when the mothers were supplemented during the last 5 days of gestation and during lactation, highlighted by a decrease in the age of pigs arriving at the slaughterhouse meaning an accelerated growth in post-weaning period (Apper et al. 2016). Stimulation of immune defenses of piglets through the maternal scFOS supplementation may explain these positive results on the reduction in the mortality rate and the improvement of growth, as previously demonstrated (Le Bourgot et al. 2014; Le Bourgot et al. 2017).

A direct supplementation of scFOS to piglets during the lactation period also increased body weight of piglets and decreased post-weaning mortality rate (Ayuso et al. 2020). The intake of scFOS after weaning may also directly improve body weight, as demonstrated by an increase in ADG and a decrease in feed-to-gain ratio following one month of scFOS supplementation (Zhao et al. 2019). However, the window of supplementation with scFOS seems crucial to maximize the health benefits, such as immune maturation and growth performances. Indeed, less pronounced effects have been observed when scFOS were administered directly during lactation vs perinatal supplementation (Ayuso et al. 2020). Additionally, others studies demonstrated that maternal scFOS supplementation represents the best strategy to stimulate immune system maturation in lactation and improve gut defenses and growth after weaning (Le Bourgot et al. 2014; Apper et al. 2016; Le Bourgot et al. 2016; Le Bourgot et al. 2017) in comparison to direct scFOS supplementation after weaning. It may be explained by the impact of scFOS in sow diet on the composition of colostrum as well as on the early colonization of the gut microbiota, as observed in the current study. Positive correlations between some bacterial groups and piglet WG have been established in the study of Mach et al. suggesting that the composition of the intestinal microbiota during neonatal life may be involved in the establishment of the piglet’s defenses ensuring better growth performances (Mach et al. 2015).

In our study, some differential effects were observed between both supplemented groups low scFOS and mid scFOS. Interestingly, we demonstrated that a very low dose of scFOS in the maternal diet (0.15% in gestation, followed by 0.10% in lactation) was efficient to improve the composition of colostrum and to modulate the gut microbiota composition in early life with similar subsequent consequences on growth after weaning than the highest dose of scFOS. Sow nutrition around farrowing, therefore, represents an effective strategy to promote the growth and health of piglets in the short and long term.

## Conclusion

Altogether, these results demonstrated that scFOS prebiotic supplementation during the perinatal period improved the nutritional and immune quality of colostrum, although overall microbial community structure was not altered in sow feces. Nevertheless, sow scFOS supplementation induced targeted changes in the early establishment of the piglet intestinal microbiota, including increases in several Lactobacillus-related genera and decreases in selected members of Spirochaetota and Bacteroidota on 2 d of age. These early modulations appear to be beneficial in promoting adaptation during the weaning transition, as low and moderate scFOS supplementation both improved post-weaning feed efficiency.

## Supplementary Material

skag140_Supplementary_Data

## Data Availability

The data underlying this article will be shared on reasonable request to the corresponding author.

## Abbreviations

ADFI: average daily feed intake
ADG: average daily gain
ASV: amplicon sequence variants
BF: back fat
BiW: body weight at birth
BW: body weight
CI: colostrum intake
EE: ether extract
FCR: feed conversion ratio
ME: metabolizable energy
N: nitrogen
NEFA: non-esterified fatty acids
SCFA: short-chain fatty acids
scFOS: short-chain fructo-oligosaccharides
TS: time suckling
WG: weight gain
