# Supplementary material for: Perinatal short-chain fructo-oligosaccharide supplementation to sow affects colostrum quality, and consequently microbiota composition and performances of progeny
Source: J Anim Sci. 2026 May 9;104:skag140. doi: 10.1093/jas/skag140 (PMC13175178; doi:10.1093/jas/skag140)
Supplement: skag140_Supplementary_Data [file skag140_supplementary_data.docx]

Supplementary Figure 1. Rarefaction curves of faecal samples (N=90). ASV (amplicon sequence variant) table was normalized to 4497 reads (sample size) per sample by single rarefaction.


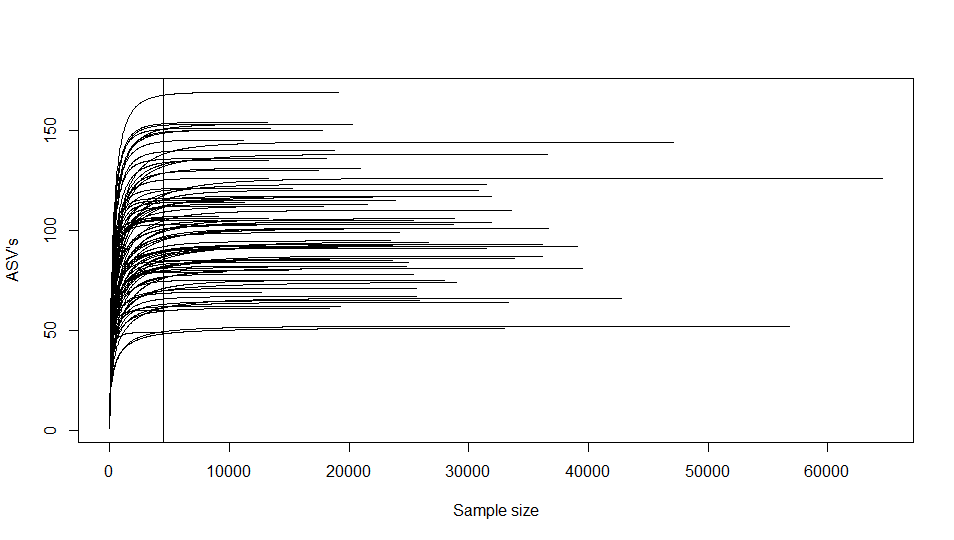


Supplementary Figure 2. Numbers of bacterial cells (log_10_) in samples of faeces in sows 2 days post-partum (n=10) and in piglets at day 2 after birth (n=20) as determined by flow cytometry.


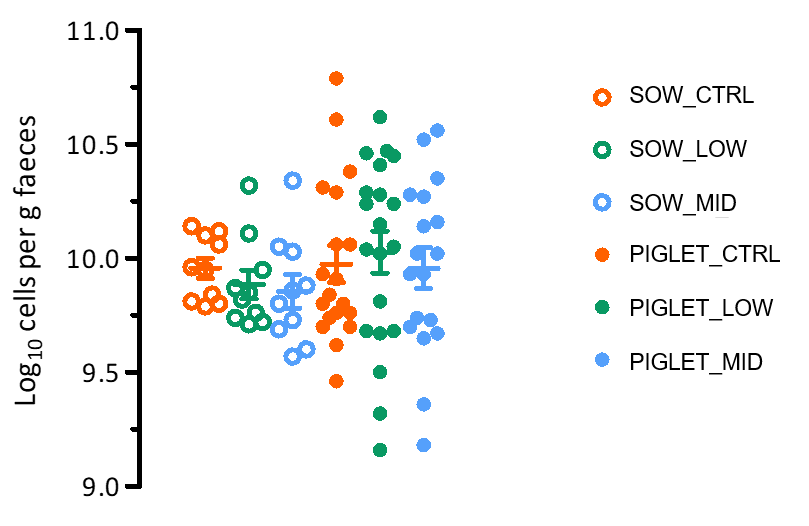


CTRL: control group; LOW: sows supplemented with 0.15 % scFOS from d28 pre-partum to d2 post-partum (gestation and transition diet) and 0.10 % during lactation (lactation diet); MID: sows supplemented with 0.33 % scFOS from d28 pre-partum to d2 post-partum (gestation and transition diet) and 0.15 % during lactation (lactation diet).

Supplementary Figure 3. Effect of treatment on Chao1 index (richness), Shannon index (evenness) and reciprocal Simpson index (diversity) (alpha diversity) at ASV and genus level in faeces in sows 2 days post-partum (n=10) (a and b, respectively) and at ASV and genus in faeces in piglets at day 2 after birth (n=20) (c and d, respectively). ns = non-significant difference, P > 0.05.

| (a)  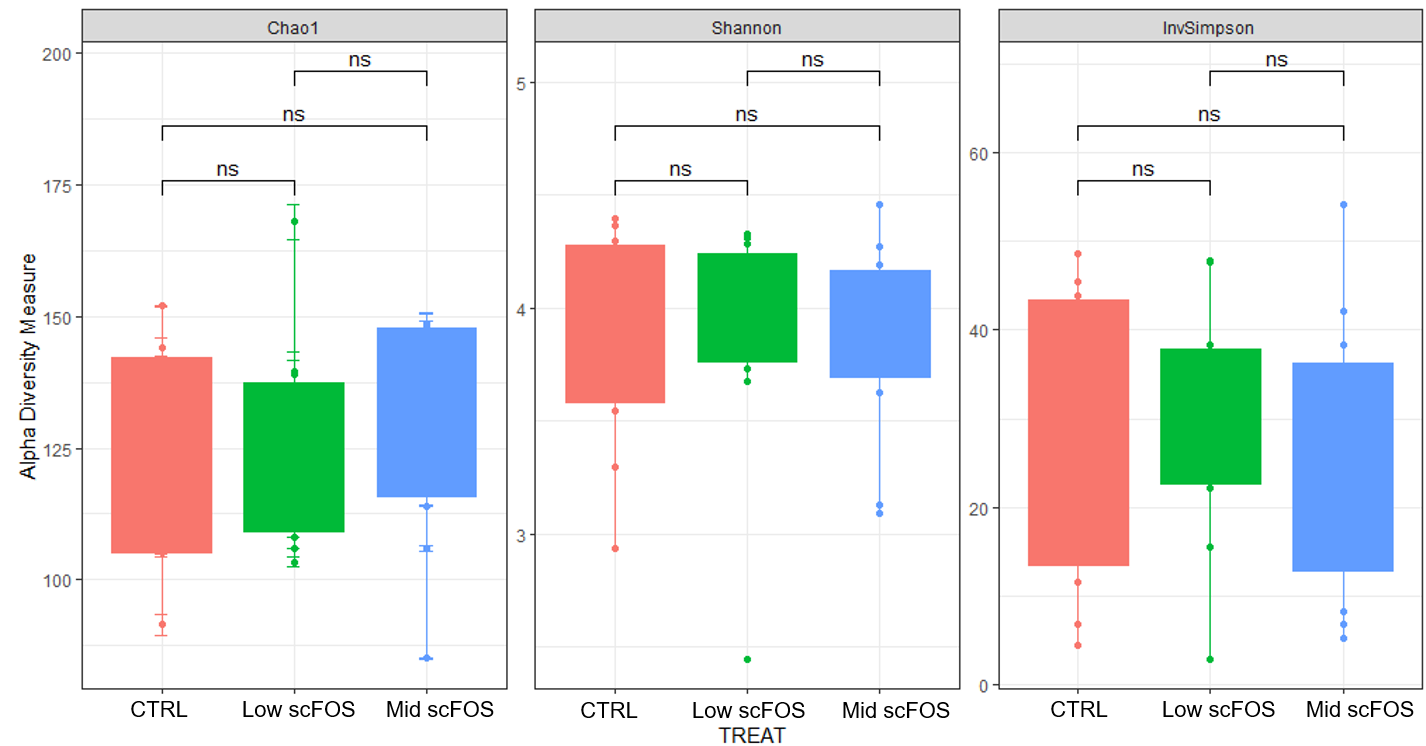 | (b)  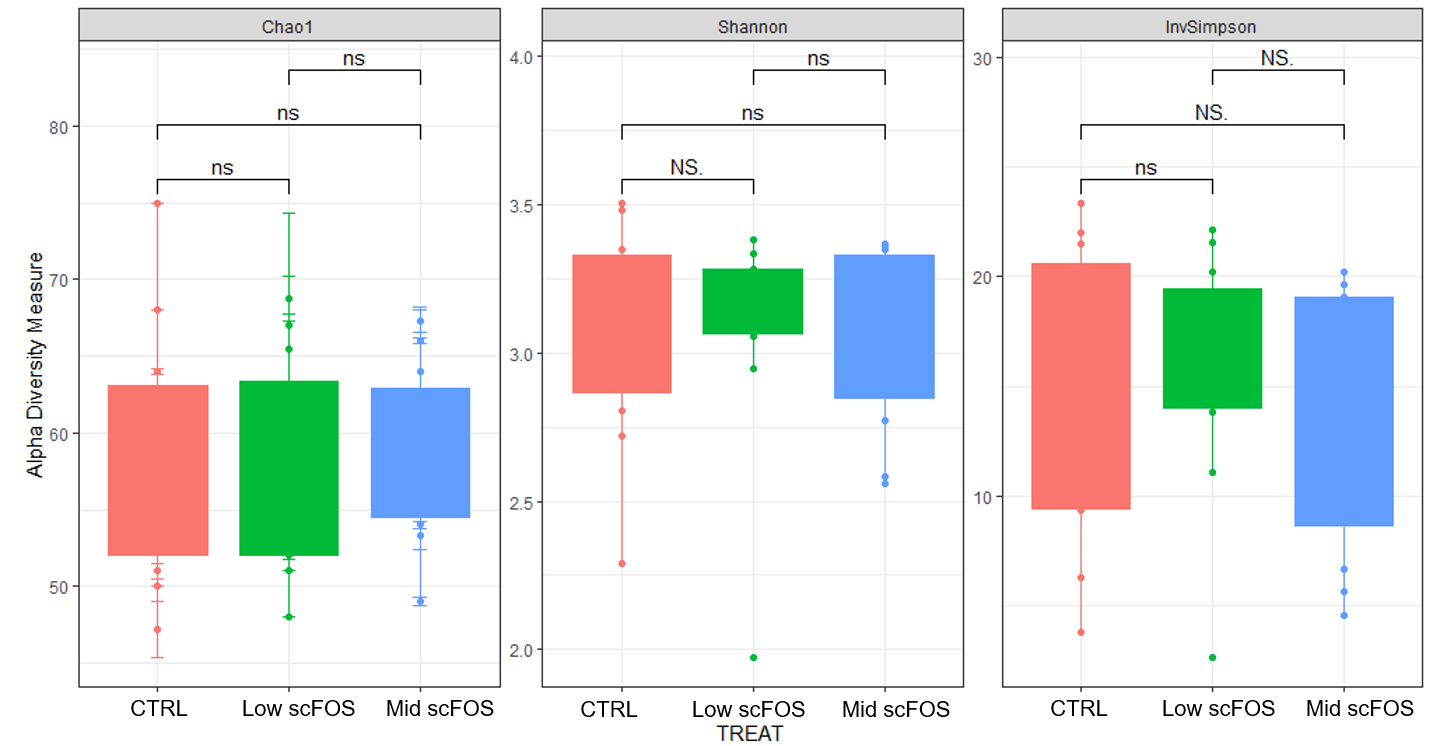 |
| --- | --- |
| (c)  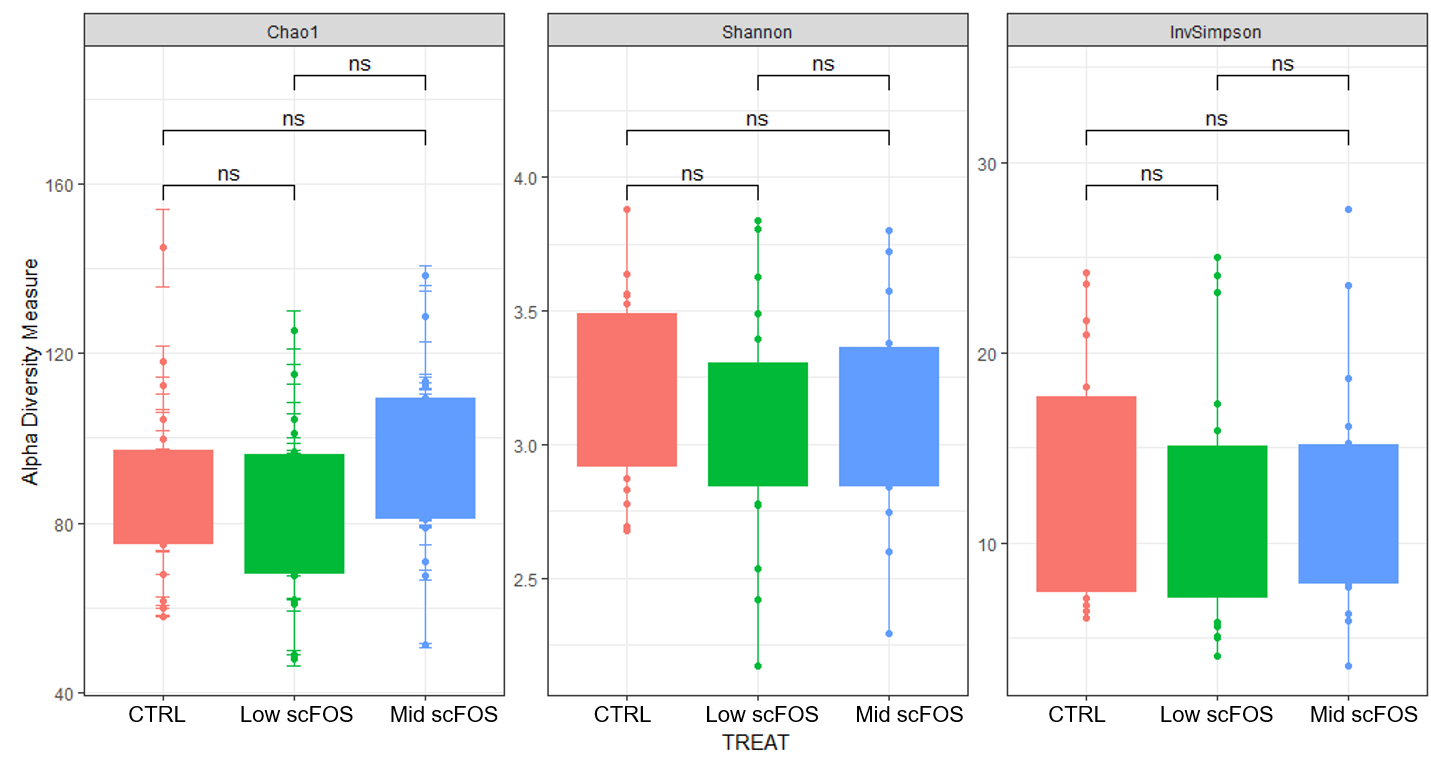 | (d)  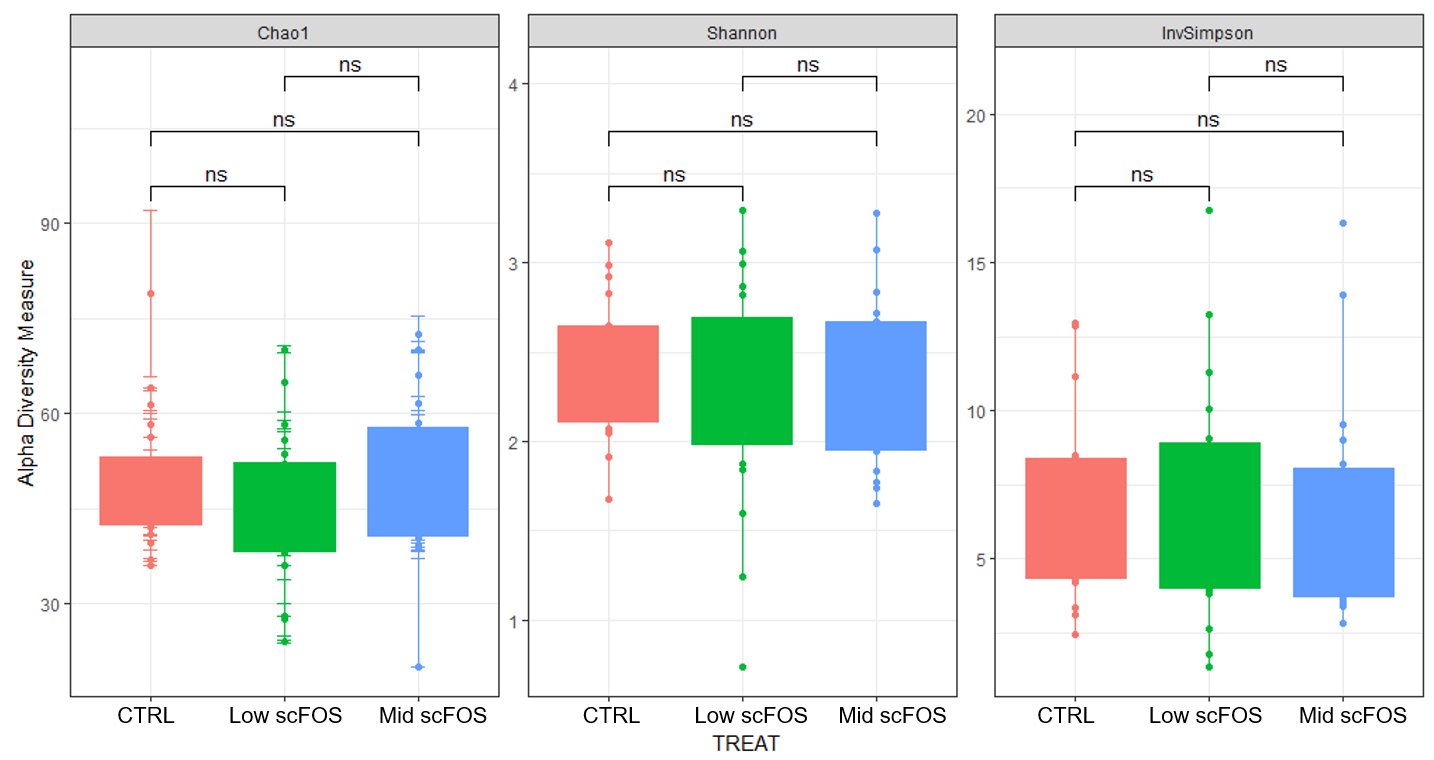 |

CTRL: control group; Low scFOS: sows supplemented with 0.15 % scFOS from d28 pre-partum to d2 post-partum (gestation and transition diet) and 0.10 % during lactation (lactation diet); Mid scFOS: sows supplemented with 0.33 % scFOS from d28 pre-partum to d2 post-partum (gestation and transition diet) and 0.15 % during lactation (lactation diet).

Supplementary table 1. Effect of dam diet on performances of piglets after weaning

| **Item** | **CTRL** | | **Low scFOS** | | **Mid scFOS** | | **P-value** |
| --- | --- | --- | --- | --- | --- | --- | --- |
|  | **Mean** | **SE** | **Mean** | **SE** | **Mean** | **SE** |  |
| BW at weaning, kg | 6.4 | 0.37 | 6.5 | 0.35 | 6.4 | 0.35 | 0.886 |
| BW at d14 post-weaning, kg | 8.1 | 0.40 | 8.1 | 0.38 | 7.9 | 0.38 | 0.637 |
| BW at d42 post-weaning, kg | 16.9 | 0.70 | 17.6 | 0.70 | 16.9 | 0.70 | 0.992 |
| ADG d0-14, g/d | 120 | 9 | 116 | 9 | 106 | 9 | 0.299 |
| ADG d14-42, g/d | 312 | 14 | 339 | 13 | 322 | 13 | 0.615 |
| ADFI d0-14, g/d | 205 | 15 | 195 | 13 | 192 | 13 | 0.539 |
| ADFI d14-42, g/d | 532 | 23 | 558 | 20 | 518 | 20 | 0.638 |
| FCR d0-14, g/g | 1.79 | 0.11 | 1.71 | 0.10 | 1.95 | 0.10 | 0.250 |
| FCR d14-42, g/g | 1.74^b^ | 0.03 | 1.65^a^ | 0.03 | 1.64^a^ | 0.03 | 0.023 |

BW: Body Weight; D: Day with weaning as reference day 0; ADG: average daily gain; ADFI: average daily feed intake; FCR: feed conversion ratio; CTRL: control group; Low scFOS: group supplemented with the lowest dose of scFOS; Mid scFOS: group supplemented with the highest dose of scFOS. Treatments without common letter are significantly different at P < 0.05.

Supplementary Table 2. Effect of treatment on relative abundance (%) of bacterial taxa in faeces in sows 2 days post-partum (n=10/group)^a^. At phylum, all taxa are given, for other taxa levels groups with overall relative abundance >0.05% are shown.

| **Taxa level** | **CTRL** | **Low scFOS** | **Mid scFOS** |
| --- | --- | --- | --- |
| **Phylum** |  |  |  |
| p_Spirochaetota | 3.144 | 2.135 | 1.890 |
| p_Proteobacteria | 0.805 | 0.543 | 1.012 |
| p_Planctomycetota | 0.429 | 0.578 | 0.207 |
| p_Patescibacteria | 0.305 | 0.445 | 0.280 |
| p_NA | 0.218 | 0.120 | 0.071 |
| p_Fusobacteriota | 0.269 | 0.002 | 0.000 |
| p_Firmicutes | 70.336 | 74.292 | 74.049 |
| p_Desulfobacterota | 0.789 | 0.943 | 0.941 |
| p_Bacteroidota | 23.280 | 20.683 | 21.276 |
| p_Actinobacteriota | 0.425 | 0.260 | 0.274 |
| **Family** |  |  |  |
| p_Actinobacteriota_c_Coriobacteriia_o_Coriobacteriales_f_Atopobiaceae | 0.111 | 0.033 | 0.120 |
| p_Actinobacteriota_c_Coriobacteriia_o_Coriobacteriales_f_Coriobacteriaceae | 0.158 | 0.024 | 0.011 |
| p_Actinobacteriota_c_Coriobacteriia_o_Coriobacteriales_f_Eggerthellaceae | 0.085 | 0.069 | 0.042 |
| p_Actinobacteriota_c_Coriobacteriia_o_Coriobacteriales_f_NA | 0.064 | 0.133 | 0.100 |
| p_Bacteroidota_c_Bacteroidia_o_Bacteroidales_f_Bacteroidaceae | 2.860 | 0.656 | 0.809 |
| p_Bacteroidota_c_Bacteroidia_o_Bacteroidales_f_Bacteroidales BS11 gut group | 0.843 | 0.018 | 1.950 |
| p_Bacteroidota_c_Bacteroidia_o_Bacteroidales_f_F082 | 0.334 | 0.338 | 0.489 |
| p_Bacteroidota_c_Bacteroidia_o_Bacteroidales_f_Muribaculaceae | 4.688 | 4.425 | 4.425 |
| p_Bacteroidota_c_Bacteroidia_o_Bacteroidales_f_p-251-o5 | 0.120 | 0.127 | 0.100 |
| p_Bacteroidota_c_Bacteroidia_o_Bacteroidales_f_p-2534-18B5 gut group | 0.712 | 1.261 | 1.401 |
| p_Bacteroidota_c_Bacteroidia_o_Bacteroidales_f_Paludibacteraceae | 0.169 | 0.191 | 0.091 |
| p_Bacteroidota_c_Bacteroidia_o_Bacteroidales_f_Prevotellaceae | 9.480 | 10.489 | 8.575 |
| p_Bacteroidota_c_Bacteroidia_o_Bacteroidales_f_Rikenellaceae | 4.072 | 3.173 | 3.436 |
| p_Desulfobacterota_c_Desulfovibrionia_o_Desulfovibrionales_f_Desulfovibrionaceae | 0.752 | 0.841 | 0.901 |
| p_Desulfobacterota_c_Desulfuromonadia_o_Bradymonadales_f_NA | 0.038 | 0.102 | 0.040 |
| p_Firmicutes_c_Bacilli_o_Erysipelotrichales_f_Erysipelatoclostridiaceae | 0.507 | 0.282 | 1.323 |
| p_Firmicutes_c_Bacilli_o_Erysipelotrichales_f_Erysipelotrichaceae | 1.892 | 2.824 | 3.376 |
| p_Firmicutes_c_Bacilli_o_Lactobacillales_f_Lactobacillaceae | 15.361 | 18.624 | 20.781 |
| p_Firmicutes_c_Bacilli_o_Lactobacillales_f_Streptococcaceae | 0.589 | 0.058 | 0.336 |
| p_Firmicutes_c_Bacilli_o_RF39_f_NA | 1.439 | 1.265 | 0.603 |
| p_Firmicutes_c_Clostridia_o_Christensenellales_f_Christensenellaceae | 5.906 | 5.751 | 4.665 |
| p_Firmicutes_c_Clostridia_o_Clostridia UCG-014_f_NA | 4.852 | 2.897 | 2.904 |
| p_Firmicutes_c_Clostridia_o_Clostridiales_f_Clostridiaceae | 9.435 | 10.374 | 7.928 |
| p_Firmicutes_c_Clostridia_o_Lachnospirales_f_Lachnospiraceae | 7.714 | 7.074 | 6.344 |
| p_Firmicutes_c_Clostridia_o_Oscillospirales_f_[Eubacterium] coprostanoligenes group | 2.900 | 2.508 | 3.502 |
| p_Firmicutes_c_Clostridia_o_Oscillospirales_f_Oscillospiraceae | 7.814 | 8.495 | 7.776 |
| p_Firmicutes_c_Clostridia_o_Oscillospirales_f_Ruminococcaceae | 2.811 | 2.644 | 2.628 |
| p_Firmicutes_c_Clostridia_o_Oscillospirales_f_UCG-010 | 0.294 | 0.249 | 0.236 |
| p_Firmicutes_c_Clostridia_o_Peptostreptococcales-Tissierellales_f_Anaerovoracaceae | 1.239 | 1.067 | 1.103 |
| p_Firmicutes_c_Clostridia_o_Peptostreptococcales-Tissierellales_f_Peptostreptococcaceae | 6.449 | 9.397 | 9.004 |
| p_Firmicutes_c_Negativicutes_o_Acidaminococcales_f_Acidaminococcaceae | 0.703 | 0.720 | 1.452 |
| p_Firmicutes_c_Negativicutes_o_Veillonellales-Selenomonadales_f_Selenomonadaceae | 0.053 | 0.036 | 0.087 |
| p_Firmicutes_c_Negativicutes_o_Veillonellales-Selenomonadales_f_Veillonellaceae | 0.378 | 0.027 | 0.000 |
| p_Fusobacteriota_c_Fusobacteriia_o_Fusobacteriales_f_Fusobacteriaceae | 0.269 | 0.002 | 0.000 |
| p_NA_c_NA_o_NA_f_NA | 0.218 | 0.120 | 0.071 |
| p_Patescibacteria_c_Saccharimonadia_o_Saccharimonadales_f_Saccharimonadaceae | 0.305 | 0.445 | 0.280 |
| p_Planctomycetota_c_Planctomycetes_o_Pirellulales_f_Pirellulaceae | 0.429 | 0.578 | 0.207 |
| p_Proteobacteria_c_Gammaproteobacteria_o_Enterobacterales_f_Enterobacteriaceae | 0.796 | 0.543 | 1.012 |
| p_Spirochaetota_c_Spirochaetia_o_Spirochaetales_f_Spirochaetaceae | 3.144 | 2.135 | 1.890 |
| **Genus** |  |  |  |
| p_Actinobacteriota_c_Coriobacteriia_o_Coriobacteriales_f_Atopobiaceae_g_NA | 0.111 | 0.033 | 0.120 |
| p_Actinobacteriota_c_Coriobacteriia_o_Coriobacteriales_f_Coriobacteriaceae_g_Collinsella | 0.158 | 0.024 | 0.011 |
| p_Actinobacteriota_c_Coriobacteriia_o_Coriobacteriales_f_Eggerthellaceae_g_NA | 0.069 | 0.053 | 0.033 |
| p_Actinobacteriota_c_Coriobacteriia_o_Coriobacteriales_f_NA_g_NA | 0.064 | 0.133 | 0.100 |
| p_Bacteroidota_c_Bacteroidia_o_Bacteroidales_f_Bacteroidaceae_g_Bacteroides | 2.860 | 0.656 | 0.809 |
| p_Bacteroidota_c_Bacteroidia_o_Bacteroidales_f_Bacteroidales BS11 gut group_g_NA | 0.843 | 0.018 | 1.950 |
| p_Bacteroidota_c_Bacteroidia_o_Bacteroidales_f_F082_g_NA | 0.334 | 0.338 | 0.489 |
| p_Bacteroidota_c_Bacteroidia_o_Bacteroidales_f_Muribaculaceae_g_NA | 4.652 | 4.401 | 4.425 |
| p_Bacteroidota_c_Bacteroidia_o_Bacteroidales_f_p-251-o5_g_NA | 0.120 | 0.127 | 0.100 |
| p_Bacteroidota_c_Bacteroidia_o_Bacteroidales_f_p-2534-18B5 gut group_g_NA | 0.712 | 1.261 | 1.401 |
| p_Bacteroidota_c_Bacteroidia_o_Bacteroidales_f_Paludibacteraceae_g_NA | 0.169 | 0.191 | 0.091 |
| p_Bacteroidota_c_Bacteroidia_o_Bacteroidales_f_Prevotellaceae_g_Alloprevotella | 0.193 | 0.329 | 0.247 |
| p_Bacteroidota_c_Bacteroidia_o_Bacteroidales_f_Prevotellaceae_g_NA | 0.042 | 0.116 | 0.024 |
| p_Bacteroidota_c_Bacteroidia_o_Bacteroidales_f_Prevotellaceae_g_Prevotella | 0.423 | 0.274 | 0.336 |
| p_Bacteroidota_c_Bacteroidia_o_Bacteroidales_f_Prevotellaceae_g_Prevotella_7 | 0.129 | 0.078 | 0.193 |
| p_Bacteroidota_c_Bacteroidia_o_Bacteroidales_f_Prevotellaceae_g_Prevotella_9 | 0.894 | 0.509 | 0.714 |
| p_Bacteroidota_c_Bacteroidia_o_Bacteroidales_f_Prevotellaceae_g_Prevotellaceae NK3B31 group | 4.227 | 5.139 | 4.492 |
| p_Bacteroidota_c_Bacteroidia_o_Bacteroidales_f_Prevotellaceae_g_Prevotellaceae UCG-001 | 3.035 | 3.540 | 2.208 |
| p_Bacteroidota_c_Bacteroidia_o_Bacteroidales_f_Prevotellaceae_g_Prevotellaceae UCG-003 | 0.511 | 0.420 | 0.294 |
| p_Bacteroidota_c_Bacteroidia_o_Bacteroidales_f_Prevotellaceae_g_Prevotellaceae UCG-004 | 0.024 | 0.085 | 0.067 |
| p_Bacteroidota_c_Bacteroidia_o_Bacteroidales_f_Rikenellaceae_g_dgA-11 gut group | 0.053 | 0.049 | 0.064 |
| p_Bacteroidota_c_Bacteroidia_o_Bacteroidales_f_Rikenellaceae_g_Rikenellaceae RC9 gut group | 4.018 | 3.124 | 3.371 |
| p_Desulfobacterota_c_Desulfovibrionia_o_Desulfovibrionales_f_Desulfovibrionaceae_g_Desulfovibrio | 0.752 | 0.841 | 0.901 |
| p_Desulfobacterota_c_Desulfuromonadia_o_Bradymonadales_f_NA_g_NA | 0.038 | 0.102 | 0.040 |
| p_Firmicutes_c_Bacilli_o_Erysipelotrichales_f_Erysipelatoclostridiaceae_g_Asteroleplasma | 0.242 | 0.060 | 0.905 |
| p_Firmicutes_c_Bacilli_o_Erysipelotrichales_f_Erysipelatoclostridiaceae_g_Catenibacterium | 0.113 | 0.189 | 0.091 |
| p_Firmicutes_c_Bacilli_o_Erysipelotrichales_f_Erysipelatoclostridiaceae_g_UCG-004 | 0.136 | 0.033 | 0.316 |
| p_Firmicutes_c_Bacilli_o_Erysipelotrichales_f_Erysipelotrichaceae_g_NA | 0.040 | 0.100 | 0.078 |
| p_Firmicutes_c_Bacilli_o_Erysipelotrichales_f_Erysipelotrichaceae_g_Turicibacter | 1.772 | 2.626 | 3.235 |
| p_Firmicutes_c_Bacilli_o_Lactobacillales_f_Lactobacillaceae_g_HT002 | 3.138 | 4.479 | 3.927 |
| p_Firmicutes_c_Bacilli_o_Lactobacillales_f_Lactobacillaceae_g_Lactobacillus | 11.545 | 13.689 | 16.131 |
| p_Firmicutes_c_Bacilli_o_Lactobacillales_f_Lactobacillaceae_g_Limosilactobacillus | 0.678 | 0.456 | 0.723 |
| p_Firmicutes_c_Bacilli_o_Lactobacillales_f_Streptococcaceae_g_Streptococcus | 0.589 | 0.058 | 0.336 |
| p_Firmicutes_c_Bacilli_o_RF39_f_NA_g_NA | 1.439 | 1.265 | 0.603 |
| p_Firmicutes_c_Clostridia_o_Christensenellales_f_Christensenellaceae_g_Christensenellaceae R-7 group | 5.348 | 5.464 | 4.503 |
| p_Firmicutes_c_Clostridia_o_Christensenellales_f_Christensenellaceae_g_NA | 0.558 | 0.287 | 0.162 |
| p_Firmicutes_c_Clostridia_o_Clostridia UCG-014_f_NA_g_NA | 4.852 | 2.897 | 2.904 |
| p_Firmicutes_c_Clostridia_o_Clostridiales_f_Clostridiaceae_g_Clostridium sensu stricto 1 | 6.191 | 7.663 | 6.382 |
| p_Firmicutes_c_Clostridia_o_Clostridiales_f_Clostridiaceae_g_Sarcina | 3.244 | 2.711 | 1.545 |
| p_Firmicutes_c_Clostridia_o_Lachnospirales_f_Lachnospiraceae_g_[Eubacterium] hallii group | 0.247 | 0.469 | 0.411 |
| p_Firmicutes_c_Clostridia_o_Lachnospirales_f_Lachnospiraceae_g_[Eubacterium] ruminantium group | 0.216 | 0.089 | 0.176 |
| p_Firmicutes_c_Clostridia_o_Lachnospirales_f_Lachnospiraceae_g_[Ruminococcus] torques group | 0.105 | 0.020 | 0.033 |
| p_Firmicutes_c_Clostridia_o_Lachnospirales_f_Lachnospiraceae_g_Blautia | 0.085 | 0.118 | 0.169 |
| p_Firmicutes_c_Clostridia_o_Lachnospirales_f_Lachnospiraceae_g_Coprococcus | 0.280 | 0.098 | 0.251 |
| p_Firmicutes_c_Clostridia_o_Lachnospirales_f_Lachnospiraceae_g_Lachnospira | 0.098 | 0.051 | 0.093 |
| p_Firmicutes_c_Clostridia_o_Lachnospirales_f_Lachnospiraceae_g_Lachnospiraceae AC2044 group | 1.728 | 1.775 | 1.517 |
| p_Firmicutes_c_Clostridia_o_Lachnospirales_f_Lachnospiraceae_g_Lachnospiraceae NK3A20 group | 0.178 | 0.051 | 0.004 |
| p_Firmicutes_c_Clostridia_o_Lachnospirales_f_Lachnospiraceae_g_Lachnospiraceae NK4A136 group | 0.998 | 0.907 | 0.649 |
| p_Firmicutes_c_Clostridia_o_Lachnospirales_f_Lachnospiraceae_g_Lachnospiraceae UCG-007 | 0.102 | 0.036 | 0.145 |
| p_Firmicutes_c_Clostridia_o_Lachnospirales_f_Lachnospiraceae_g_Lachnospiraceae UCG-009 | 0.327 | 0.198 | 0.254 |
| p_Firmicutes_c_Clostridia_o_Lachnospirales_f_Lachnospiraceae_g_Lachnospiraceae XPB1014 group | 2.579 | 2.711 | 2.021 |
| p_Firmicutes_c_Clostridia_o_Lachnospirales_f_Lachnospiraceae_g_Marvinbryantia | 0.140 | 0.249 | 0.240 |
| p_Firmicutes_c_Clostridia_o_Lachnospirales_f_Lachnospiraceae_g_NA | 0.116 | 0.165 | 0.182 |
| p_Firmicutes_c_Clostridia_o_Lachnospirales_f_Lachnospiraceae_g_Oribacterium | 0.211 | 0.105 | 0.129 |
| p_Firmicutes_c_Clostridia_o_Lachnospirales_f_Lachnospiraceae_g_Shuttleworthia | 0.258 | 0.000 | 0.027 |
| p_Firmicutes_c_Clostridia_o_Oscillospirales_f_[Eubacterium] coprostanoligenes group_g_NA | 2.900 | 2.508 | 3.502 |
| p_Firmicutes_c_Clostridia_o_Oscillospirales_f_Oscillospiraceae_g_NA | 0.260 | 0.254 | 0.191 |
| p_Firmicutes_c_Clostridia_o_Oscillospirales_f_Oscillospiraceae_g_NK4A214 group | 1.810 | 1.986 | 2.090 |
| p_Firmicutes_c_Clostridia_o_Oscillospirales_f_Oscillospiraceae_g_UCG-002 | 3.814 | 4.272 | 3.847 |
| p_Firmicutes_c_Clostridia_o_Oscillospirales_f_Oscillospiraceae_g_UCG-005 | 1.883 | 1.928 | 1.610 |
| p_Firmicutes_c_Clostridia_o_Oscillospirales_f_Ruminococcaceae_g_[Eubacterium] siraeum group | 0.173 | 0.131 | 0.185 |
| p_Firmicutes_c_Clostridia_o_Oscillospirales_f_Ruminococcaceae_g_Candidatus Soleaferrea | 0.167 | 0.162 | 0.254 |
| p_Firmicutes_c_Clostridia_o_Oscillospirales_f_Ruminococcaceae_g_Faecalibacterium | 0.100 | 0.178 | 0.133 |
| p_Firmicutes_c_Clostridia_o_Oscillospirales_f_Ruminococcaceae_g_NA | 0.271 | 0.185 | 0.249 |
| p_Firmicutes_c_Clostridia_o_Oscillospirales_f_Ruminococcaceae_g_Ruminococcus | 1.912 | 1.768 | 1.557 |
| p_Firmicutes_c_Clostridia_o_Oscillospirales_f_Ruminococcaceae_g_Subdoligranulum | 0.167 | 0.173 | 0.238 |
| p_Firmicutes_c_Clostridia_o_Oscillospirales_f_UCG-010_g_NA | 0.294 | 0.249 | 0.236 |
| p_Firmicutes_c_Clostridia_o_Peptostreptococcales-Tissierellales_f_Anaerovoracaceae_g_Family XIII AD3011 group | 1.216 | 1.067 | 1.085 |
| p_Firmicutes_c_Clostridia_o_Peptostreptococcales-Tissierellales_f_Peptostreptococcaceae_g_Intestinibacter | 0.133 | 0.131 | 0.222 |
| p_Firmicutes_c_Clostridia_o_Peptostreptococcales-Tissierellales_f_Peptostreptococcaceae_g_Romboutsia | 2.871 | 3.849 | 4.263 |
| p_Firmicutes_c_Clostridia_o_Peptostreptococcales-Tissierellales_f_Peptostreptococcaceae_g_Terrisporobacter | 3.445 | 5.417 | 4.519 |
| p_Firmicutes_c_Negativicutes_o_Acidaminococcales_f_Acidaminococcaceae_g_Phascolarctobacterium | 0.703 | 0.720 | 1.452 |
| p_Firmicutes_c_Negativicutes_o_Veillonellales-Selenomonadales_f_Selenomonadaceae_g_Anaerovibrio | 0.053 | 0.036 | 0.087 |
| p_Firmicutes_c_Negativicutes_o_Veillonellales-Selenomonadales_f_Veillonellaceae_g_Megasphaera | 0.378 | 0.027 | 0.000 |
| p_Fusobacteriota_c_Fusobacteriia_o_Fusobacteriales_f_Fusobacteriaceae_g_Fusobacterium | 0.269 | 0.002 | 0.000 |
| p_NA_c_NA_o_NA_f_NA_g_NA | 0.218 | 0.120 | 0.071 |
| p_Patescibacteria_c_Saccharimonadia_o_Saccharimonadales_f_Saccharimonadaceae_g_Candidatus Saccharimonas | 0.305 | 0.445 | 0.280 |
| p_Planctomycetota_c_Planctomycetes_o_Pirellulales_f_Pirellulaceae_g_CPla-4 termite group | 0.345 | 0.438 | 0.027 |
| p_Planctomycetota_c_Planctomycetes_o_Pirellulales_f_Pirellulaceae_g_p-1088-a5 gut group | 0.085 | 0.140 | 0.180 |
| p_Proteobacteria_c_Gammaproteobacteria_o_Enterobacterales_f_Enterobacteriaceae_g_Escherichia-Shigella | 0.796 | 0.543 | 1.012 |
| **p_Spirochaetota_c_Spirochaetia_o_Spirochaetales_f_Spirochaetaceae_g_Sphaerochaeta** | **0.156^a^** | **0.020^b^** | **0.207^a^** |
| p_Spirochaetota_c_Spirochaetia_o_Spirochaetales_f_Spirochaetaceae_g_Treponema | 2.989 | 2.115 | 1.683 |
| **Species** |  |  |  |
| p_Actinobacteriota_c_Coriobacteriia_o_Coriobacteriales_f_Atopobiaceae_g_NA_s_NA | 0.111 | 0.033 | 0.120 |
| p_Actinobacteriota_c_Coriobacteriia_o_Coriobacteriales_f_Coriobacteriaceae_g_Collinsella_s_NA | 0.153 | 0.024 | 0.011 |
| p_Actinobacteriota_c_Coriobacteriia_o_Coriobacteriales_f_Eggerthellaceae_g_NA_s_NA | 0.069 | 0.053 | 0.033 |
| p_Actinobacteriota_c_Coriobacteriia_o_Coriobacteriales_f_NA_g_NA_s_NA | 0.064 | 0.133 | 0.100 |
| p_Bacteroidota_c_Bacteroidia_o_Bacteroidales_f_Bacteroidaceae_g_Bacteroides_s_fragilis | 0.173 | 0.000 | 0.247 |
| p_Bacteroidota_c_Bacteroidia_o_Bacteroidales_f_Bacteroidaceae_g_Bacteroides_s_NA | 1.679 | 0.625 | 0.467 |
| p_Bacteroidota_c_Bacteroidia_o_Bacteroidales_f_Bacteroidaceae_g_Bacteroides_s_plebeius | 0.916 | 0.029 | 0.056 |
| p_Bacteroidota_c_Bacteroidia_o_Bacteroidales_f_Bacteroidales BS11 gut group_g_NA_s_NA | 0.843 | 0.018 | 1.950 |
| p_Bacteroidota_c_Bacteroidia_o_Bacteroidales_f_F082_g_NA_s_NA | 0.334 | 0.338 | 0.489 |
| p_Bacteroidota_c_Bacteroidia_o_Bacteroidales_f_Muribaculaceae_g_NA_s_NA | 4.652 | 4.401 | 4.425 |
| p_Bacteroidota_c_Bacteroidia_o_Bacteroidales_f_p-251-o5_g_NA_s_NA | 0.120 | 0.127 | 0.100 |
| p_Bacteroidota_c_Bacteroidia_o_Bacteroidales_f_p-2534-18B5 gut group_g_NA_s_NA | 0.712 | 1.261 | 1.401 |
| p_Bacteroidota_c_Bacteroidia_o_Bacteroidales_f_Paludibacteraceae_g_NA_s_NA | 0.169 | 0.191 | 0.091 |
| p_Bacteroidota_c_Bacteroidia_o_Bacteroidales_f_Prevotellaceae_g_Alloprevotella_s_NA | 0.193 | 0.329 | 0.247 |
| p_Bacteroidota_c_Bacteroidia_o_Bacteroidales_f_Prevotellaceae_g_NA_s_NA | 0.042 | 0.116 | 0.024 |
| p_Bacteroidota_c_Bacteroidia_o_Bacteroidales_f_Prevotellaceae_g_Prevotella_7_s_NA | 0.129 | 0.078 | 0.193 |
| p_Bacteroidota_c_Bacteroidia_o_Bacteroidales_f_Prevotellaceae_g_Prevotella_9_s_NA | 0.894 | 0.509 | 0.714 |
| p_Bacteroidota_c_Bacteroidia_o_Bacteroidales_f_Prevotellaceae_g_Prevotella_s_NA | 0.423 | 0.274 | 0.336 |
| p_Bacteroidota_c_Bacteroidia_o_Bacteroidales_f_Prevotellaceae_g_Prevotellaceae NK3B31 group_s_NA | 4.227 | 5.139 | 4.492 |
| p_Bacteroidota_c_Bacteroidia_o_Bacteroidales_f_Prevotellaceae_g_Prevotellaceae UCG-001_s_NA | 3.035 | 3.540 | 2.208 |
| p_Bacteroidota_c_Bacteroidia_o_Bacteroidales_f_Prevotellaceae_g_Prevotellaceae UCG-003_s_NA | 0.511 | 0.420 | 0.294 |
| p_Bacteroidota_c_Bacteroidia_o_Bacteroidales_f_Prevotellaceae_g_Prevotellaceae UCG-004_s_NA | 0.024 | 0.085 | 0.067 |
| p_Bacteroidota_c_Bacteroidia_o_Bacteroidales_f_Rikenellaceae_g_dgA-11 gut group_s_NA | 0.053 | 0.049 | 0.064 |
| p_Bacteroidota_c_Bacteroidia_o_Bacteroidales_f_Rikenellaceae_g_Rikenellaceae RC9 gut group_s_NA | 4.018 | 3.124 | 3.371 |
| p_Desulfobacterota_c_Desulfovibrionia_o_Desulfovibrionales_f_Desulfovibrionaceae_g_Desulfovibrio_s_NA | 0.667 | 0.687 | 0.805 |
| p_Desulfobacterota_c_Desulfovibrionia_o_Desulfovibrionales_f_Desulfovibrionaceae_g_Desulfovibrio_s_piger | 0.085 | 0.153 | 0.096 |
| p_Desulfobacterota_c_Desulfuromonadia_o_Bradymonadales_f_NA_g_NA_s_NA | 0.038 | 0.102 | 0.040 |
| p_Firmicutes_c_Bacilli_o_Erysipelotrichales_f_Erysipelatoclostridiaceae_g_Asteroleplasma_s_anaerobium | 0.116 | 0.038 | 0.189 |
| p_Firmicutes_c_Bacilli_o_Erysipelotrichales_f_Erysipelatoclostridiaceae_g_Asteroleplasma_s_NA | 0.127 | 0.022 | 0.716 |
| p_Firmicutes_c_Bacilli_o_Erysipelotrichales_f_Erysipelatoclostridiaceae_g_Catenibacterium_s_NA | 0.113 | 0.189 | 0.091 |
| p_Firmicutes_c_Bacilli_o_Erysipelotrichales_f_Erysipelatoclostridiaceae_g_UCG-004_s_NA | 0.136 | 0.033 | 0.316 |
| p_Firmicutes_c_Bacilli_o_Erysipelotrichales_f_Erysipelotrichaceae_g_NA_s_NA | 0.040 | 0.100 | 0.078 |
| p_Firmicutes_c_Bacilli_o_Erysipelotrichales_f_Erysipelotrichaceae_g_Turicibacter_s_NA | 1.772 | 2.626 | 3.235 |
| p_Firmicutes_c_Bacilli_o_Lactobacillales_f_Lactobacillaceae_g_HT002_s_NA | 3.138 | 4.479 | 3.927 |
| p_Firmicutes_c_Bacilli_o_Lactobacillales_f_Lactobacillaceae_g_Lactobacillus_s_amylovorus | 8.973 | 10.878 | 12.837 |
| p_Firmicutes_c_Bacilli_o_Lactobacillales_f_Lactobacillaceae_g_Lactobacillus_s_delbrueckii | 0.400 | 0.196 | 0.351 |
| p_Firmicutes_c_Bacilli_o_Lactobacillales_f_Lactobacillaceae_g_Lactobacillus_s_johnsonii | 2.070 | 2.446 | 2.717 |
| p_Firmicutes_c_Bacilli_o_Lactobacillales_f_Lactobacillaceae_g_Lactobacillus_s_NA | 0.102 | 0.169 | 0.225 |
| p_Firmicutes_c_Bacilli_o_Lactobacillales_f_Lactobacillaceae_g_Limosilactobacillus_s_NA | 0.678 | 0.456 | 0.723 |
| p_Firmicutes_c_Bacilli_o_Lactobacillales_f_Streptococcaceae_g_Streptococcus_s_lutetiensis | 0.474 | 0.058 | 0.298 |
| p_Firmicutes_c_Bacilli_o_RF39_f_NA_g_NA_s_NA | 1.439 | 1.265 | 0.603 |
| p_Firmicutes_c_Clostridia_o_Christensenellales_f_Christensenellaceae_g_Christensenellaceae R-7 group_s_NA | 5.348 | 5.464 | 4.503 |
| p_Firmicutes_c_Clostridia_o_Christensenellales_f_Christensenellaceae_g_NA_s_NA | 0.558 | 0.287 | 0.162 |
| p_Firmicutes_c_Clostridia_o_Clostridia UCG-014_f_NA_g_NA_s_NA | 4.852 | 2.897 | 2.904 |
| p_Firmicutes_c_Clostridia_o_Clostridiales_f_Clostridiaceae_g_Clostridium sensu stricto 1_s_baratii | 0.878 | 0.574 | 0.489 |
| p_Firmicutes_c_Clostridia_o_Clostridiales_f_Clostridiaceae_g_Clostridium sensu stricto 1_s_butyricum | 1.139 | 1.316 | 1.470 |
| p_Firmicutes_c_Clostridia_o_Clostridiales_f_Clostridiaceae_g_Clostridium sensu stricto 1_s_NA | 3.400 | 5.297 | 4.214 |
| p_Firmicutes_c_Clostridia_o_Clostridiales_f_Clostridiaceae_g_Clostridium sensu stricto 1_s_perfringens | 0.047 | 0.245 | 0.011 |
| p_Firmicutes_c_Clostridia_o_Clostridiales_f_Clostridiaceae_g_Clostridium sensu stricto 1_s_septicum | 0.727 | 0.231 | 0.198 |
| p_Firmicutes_c_Clostridia_o_Clostridiales_f_Clostridiaceae_g_Sarcina_s_NA | 3.244 | 2.711 | 1.545 |
| p_Firmicutes_c_Clostridia_o_Lachnospirales_f_Lachnospiraceae_g_[Eubacterium] hallii group_s_NA | 0.213 | 0.431 | 0.391 |
| p_Firmicutes_c_Clostridia_o_Lachnospirales_f_Lachnospiraceae_g_[Eubacterium] ruminantium group_s_NA | 0.216 | 0.089 | 0.176 |
| p_Firmicutes_c_Clostridia_o_Lachnospirales_f_Lachnospiraceae_g_Blautia_s_obeum | 0.085 | 0.118 | 0.169 |
| p_Firmicutes_c_Clostridia_o_Lachnospirales_f_Lachnospiraceae_g_Coprococcus_s_comes | 0.271 | 0.087 | 0.191 |
| p_Firmicutes_c_Clostridia_o_Lachnospirales_f_Lachnospiraceae_g_Lachnospira_s_NA | 0.098 | 0.051 | 0.093 |
| p_Firmicutes_c_Clostridia_o_Lachnospirales_f_Lachnospiraceae_g_Lachnospiraceae AC2044 group_s_NA | 1.728 | 1.775 | 1.517 |
| p_Firmicutes_c_Clostridia_o_Lachnospirales_f_Lachnospiraceae_g_Lachnospiraceae NK3A20 group_s_NA | 0.178 | 0.051 | 0.004 |
| p_Firmicutes_c_Clostridia_o_Lachnospirales_f_Lachnospiraceae_g_Lachnospiraceae NK4A136 group_s_bacterium | 0.071 | 0.082 | 0.131 |
| p_Firmicutes_c_Clostridia_o_Lachnospirales_f_Lachnospiraceae_g_Lachnospiraceae NK4A136 group_s_NA | 0.927 | 0.825 | 0.518 |
| p_Firmicutes_c_Clostridia_o_Lachnospirales_f_Lachnospiraceae_g_Lachnospiraceae UCG-007_s_NA | 0.102 | 0.036 | 0.145 |
| p_Firmicutes_c_Clostridia_o_Lachnospirales_f_Lachnospiraceae_g_Lachnospiraceae UCG-009_s_NA | 0.327 | 0.198 | 0.254 |
| p_Firmicutes_c_Clostridia_o_Lachnospirales_f_Lachnospiraceae_g_Lachnospiraceae XPB1014 group_s_NA | 2.579 | 2.711 | 2.021 |
| p_Firmicutes_c_Clostridia_o_Lachnospirales_f_Lachnospiraceae_g_Marvinbryantia_s_NA | 0.140 | 0.249 | 0.240 |
| p_Firmicutes_c_Clostridia_o_Lachnospirales_f_Lachnospiraceae_g_NA_s_NA | 0.116 | 0.165 | 0.182 |
| p_Firmicutes_c_Clostridia_o_Lachnospirales_f_Lachnospiraceae_g_Oribacterium_s_NA | 0.211 | 0.105 | 0.129 |
| p_Firmicutes_c_Clostridia_o_Lachnospirales_f_Lachnospiraceae_g_Shuttleworthia_s_NA | 0.258 | 0.000 | 0.027 |
| p_Firmicutes_c_Clostridia_o_Oscillospirales_f_[Eubacterium] coprostanoligenes group_g_NA_s_NA | 2.900 | 2.508 | 3.502 |
| p_Firmicutes_c_Clostridia_o_Oscillospirales_f_Oscillospiraceae_g_NA_s_NA | 0.260 | 0.254 | 0.191 |
| p_Firmicutes_c_Clostridia_o_Oscillospirales_f_Oscillospiraceae_g_NK4A214 group_s_NA | 1.810 | 1.986 | 2.090 |
| p_Firmicutes_c_Clostridia_o_Oscillospirales_f_Oscillospiraceae_g_UCG-002_s_NA | 3.814 | 4.272 | 3.847 |
| p_Firmicutes_c_Clostridia_o_Oscillospirales_f_Oscillospiraceae_g_UCG-005_s_NA | 1.883 | 1.928 | 1.610 |
| p_Firmicutes_c_Clostridia_o_Oscillospirales_f_Ruminococcaceae_g_[Eubacterium] siraeum group_s_NA | 0.173 | 0.131 | 0.185 |
| p_Firmicutes_c_Clostridia_o_Oscillospirales_f_Ruminococcaceae_g_Candidatus Soleaferrea_s_NA | 0.167 | 0.162 | 0.254 |
| p_Firmicutes_c_Clostridia_o_Oscillospirales_f_Ruminococcaceae_g_Faecalibacterium_s_prausnitzii | 0.100 | 0.178 | 0.133 |
| p_Firmicutes_c_Clostridia_o_Oscillospirales_f_Ruminococcaceae_g_NA_s_NA | 0.271 | 0.185 | 0.249 |
| p_Firmicutes_c_Clostridia_o_Oscillospirales_f_Ruminococcaceae_g_Ruminococcus_s_champanellensis | 0.227 | 0.467 | 0.285 |
| p_Firmicutes_c_Clostridia_o_Oscillospirales_f_Ruminococcaceae_g_Ruminococcus_s_flavefaciens | 1.643 | 0.812 | 1.263 |
| p_Firmicutes_c_Clostridia_o_Oscillospirales_f_Ruminococcaceae_g_Ruminococcus_s_NA | 0.042 | 0.489 | 0.009 |
| p_Firmicutes_c_Clostridia_o_Oscillospirales_f_Ruminococcaceae_g_Subdoligranulum_s_NA | 0.167 | 0.173 | 0.238 |
| p_Firmicutes_c_Clostridia_o_Oscillospirales_f_UCG-010_g_NA_s_NA | 0.294 | 0.249 | 0.236 |
| p_Firmicutes_c_Clostridia_o_Peptostreptococcales-Tissierellales_f_Anaerovoracaceae_g_Family XIII AD3011 group_s_NA | 1.216 | 1.067 | 1.085 |
| p_Firmicutes_c_Clostridia_o_Peptostreptococcales-Tissierellales_f_Peptostreptococcaceae_g_Intestinibacter_s_NA | 0.133 | 0.131 | 0.222 |
| p_Firmicutes_c_Clostridia_o_Peptostreptococcales-Tissierellales_f_Peptostreptococcaceae_g_Romboutsia_s_ilealis | 2.871 | 3.849 | 4.263 |
| p_Firmicutes_c_Clostridia_o_Peptostreptococcales-Tissierellales_f_Peptostreptococcaceae_g_Terrisporobacter_s_NA | 3.445 | 5.417 | 4.519 |
| p_Firmicutes_c_Negativicutes_o_Acidaminococcales_f_Acidaminococcaceae_g_Phascolarctobacterium_s_NA | 0.703 | 0.720 | 1.452 |
| p_Firmicutes_c_Negativicutes_o_Veillonellales-Selenomonadales_f_Selenomonadaceae_g_Anaerovibrio_s_NA | 0.053 | 0.036 | 0.087 |
| p_Firmicutes_c_Negativicutes_o_Veillonellales-Selenomonadales_f_Veillonellaceae_g_Megasphaera_s_elsdenii | 0.378 | 0.027 | 0.000 |
| p_Fusobacteriota_c_Fusobacteriia_o_Fusobacteriales_f_Fusobacteriaceae_g_Fusobacterium_s_mortiferum | 0.256 | 0.000 | 0.000 |
| p_NA_c_NA_o_NA_f_NA_g_NA_s_NA | 0.218 | 0.120 | 0.071 |
| p_Patescibacteria_c_Saccharimonadia_o_Saccharimonadales_f_Saccharimonadaceae_g_Candidatus Saccharimonas_s_NA | 0.305 | 0.445 | 0.280 |
| p_Planctomycetota_c_Planctomycetes_o_Pirellulales_f_Pirellulaceae_g_CPla-4 termite group_s_NA | 0.345 | 0.438 | 0.027 |
| p_Planctomycetota_c_Planctomycetes_o_Pirellulales_f_Pirellulaceae_g_p-1088-a5 gut group_s_NA | 0.085 | 0.140 | 0.180 |
| p_Proteobacteria_c_Gammaproteobacteria_o_Enterobacterales_f_Enterobacteriaceae_g_Escherichia-Shigella_s_coli | 0.796 | 0.543 | 1.012 |
| **p_Spirochaetota_c_Spirochaetia_o_Spirochaetales_f_Spirochaetaceae_g_Sphaerochaeta_s_NA** | **0.156^a^** | **0.020^b^** | **0.207^a^** |
| p_Spirochaetota_c_Spirochaetia_o_Spirochaetales_f_Spirochaetaceae_g_Treponema_s_bryantii | 0.774 | 0.465 | 0.405 |
| p_Spirochaetota_c_Spirochaetia_o_Spirochaetales_f_Spirochaetaceae_g_Treponema_s_NA | 2.166 | 1.650 | 1.276 |

^a^ Means within row without common superscript are significantly different, P < 0.05. CTRL: control group; Low scFOS: sows supplemented with 0.15 % scFOS from d28 pre-partum to d2 post-partum (gestation and transition diet) and 0.10 % during lactation (lactation diet); Mid scFOS: sows supplemented with 0.33 % scFOS from d28 pre-partum to d2 post-partum (gestation and transition diet) and 0.15 % during lactation (lactation diet).

Supplementary Table 3. Effect of treatment on relative abundance (%) of bacterial taxa in faeces in piglets at day 2 after birth (n=20/group)^a^. At phylum, all taxa are given, for other taxa levels groups with overall relative abundance >0.05% are shown.

| **Taxa level** | **CTRL** | **Low scFOS** | **Mid scFOS** |
| --- | --- | --- | --- |
| **Phylum** |  |  |  |
| p_Actinobacteriota | 3.556 | 3.167 | 1.140 |
| p_Bacteroidota | 18.905 | 14.769 | 18.493 |
| p_Desulfobacterota | 0.314 | 0.387 | 0.238 |
| p_Firmicutes | 69.189 | 72.486 | 72.432 |
| p_Fusobacteriota | 1.972 | 3.221 | 3.705 |
| p_NA | 2.686 | 0.000 | 0.105 |
| p_Patescibacteria | 0.000 | 0.000 | 0.000 |
| p_Planctomycetota | 0.034 | 0.008 | 0.011 |
| p_Proteobacteria | 2.716 | 5.913 | 3.756 |
| **p_Spirochaetota** | **0.628^a^** | **0.050^b^** | **0.120^b^** |
| **Family** |  |  |  |
| **p_Actinobacteriota_c_Actinobacteria_o_Actinomycetales_f_Actinomycetaceae** | **1.592^a^** | **1.772^a^** | **0.035^b^** |
| p_Actinobacteriota_c_Actinobacteria_o_Micrococcales_f_Micrococcaceae | 0.061 | 0.066 | 0.095 |
| p_Actinobacteriota_c_Coriobacteriia_o_Coriobacteriales_f_Atopobiaceae | 0.263 | 0.054 | 0.094 |
| p_Actinobacteriota_c_Coriobacteriia_o_Coriobacteriales_f_Coriobacteriaceae | 0.482 | 0.405 | 0.560 |
| **p_Actinobacteriota_c_Coriobacteriia_o_Coriobacteriales_f_Eggerthellaceae** | **1.157^a^** | **0.869^ab^** | **0.357^b^** |
| p_Bacteroidota_c_Bacteroidia_o_Bacteroidales_f_Bacteroidaceae | 4.858 | 4.814 | 2.486 |
| p_Bacteroidota_c_Bacteroidia_o_Bacteroidales_f_Muribaculaceae | 8.620 | 7.372 | 12.082 |
| p_Bacteroidota_c_Bacteroidia_o_Bacteroidales_f_p-2534-18B5 gut group | 0.084 | 0.000 | 0.086 |
| p_Bacteroidota_c_Bacteroidia_o_Bacteroidales_f_Prevotellaceae | 2.500 | 1.249 | 2.955 |
| p_Bacteroidota_c_Bacteroidia_o_Bacteroidales_f_Rikenellaceae | 1.553 | 0.688 | 0.591 |
| p_Bacteroidota_c_Bacteroidia_o_Bacteroidales_f_Tannerellaceae | 1.160 | 0.643 | 0.236 |
| p_Desulfobacterota_c_Desulfovibrionia_o_Desulfovibrionales_f_Desulfovibrionaceae | 0.310 | 0.275 | 0.220 |
| p_Firmicutes_c_Bacilli_o_Erysipelotrichales_f_Erysipelotrichaceae | 0.907 | 2.759 | 0.691 |
| p_Firmicutes_c_Bacilli_o_Lactobacillales_f_Enterococcaceae | 0.536 | 0.087 | 0.044 |
| p_Firmicutes_c_Bacilli_o_Lactobacillales_f_Lactobacillaceae | 31.341 | 42.115 | 43.508 |
| p_Firmicutes_c_Bacilli_o_Lactobacillales_f_Streptococcaceae | 2.661 | 2.614 | 1.156 |
| p_Firmicutes_c_Clostridia_o_Christensenellales_f_Christensenellaceae | 0.195 | 0.335 | 0.183 |
| p_Firmicutes_c_Clostridia_o_Clostridiales_f_Clostridiaceae | 3.936 | 2.668 | 2.321 |
| p_Firmicutes_c_Clostridia_o_Lachnospirales_f_Lachnospiraceae | 9.994 | 8.340 | 8.895 |
| p_Firmicutes_c_Clostridia_o_Oscillospirales_f_[Eubacterium] coprostanoligenes group | 3.352 | 3.391 | 3.683 |
| p_Firmicutes_c_Clostridia_o_Oscillospirales_f_Butyricicoccaceae | 0.121 | 0.146 | 0.493 |
| p_Firmicutes_c_Clostridia_o_Oscillospirales_f_Oscillospiraceae | 4.532 | 2.772 | 4.974 |
| p_Firmicutes_c_Clostridia_o_Oscillospirales_f_Ruminococcaceae | 1.963 | 1.443 | 1.222 |
| p_Firmicutes_c_Clostridia_o_Oscillospirales_f_UCG-010 | 0.014 | 0.107 | 0.033 |
| p_Firmicutes_c_Clostridia_o_Peptococcales_f_Peptococcaceae | 0.042 | 0.062 | 0.044 |
| p_Firmicutes_c_Clostridia_o_Peptostreptococcales-Tissierellales_f_Anaerovoracaceae | 1.761 | 1.539 | 0.783 |
| p_Firmicutes_c_Clostridia_o_Peptostreptococcales-Tissierellales_f_Peptostreptococcaceae | 1.310 | 1.624 | 2.447 |
| **p_Firmicutes_c_Negativicutes_o_Acidaminococcales_f_Acidaminococcaceae** | **5.744^a^** | **1.137^b^** | **1.230^b^** |
| p_Firmicutes_c_Negativicutes_o_Veillonellales-Selenomonadales_f_Veillonellaceae | 0.458 | 1.285 | 0.639 |
| p_Fusobacteriota_c_Fusobacteriia_o_Fusobacteriales_f_Fusobacteriaceae | 1.972 | 3.221 | 3.705 |
| p_NA_c_NA_o_NA_f_NA | 2.686 | 0.000 | 0.105 |
| p_Proteobacteria_c_Gammaproteobacteria_o_Burkholderiales_f_Oxalobacteraceae | 0.027 | 0.399 | 0.203 |
| p_Proteobacteria_c_Gammaproteobacteria_o_Enterobacterales_f_Enterobacteriaceae | 2.603 | 4.977 | 2.547 |
| p_Proteobacteria_c_Gammaproteobacteria_o_Enterobacterales_f_Pasteurellaceae | 0.085 | 0.489 | 0.969 |
| **p_Spirochaetota_c_Spirochaetia_o_Spirochaetales_f_Spirochaetaceae** | **0.628^a^** | **0.050^b^** | **0.120^b^** |
| **Genus** |  |  |  |
| **p_Actinobacteriota_c_Actinobacteria_o_Actinomycetales_f_Actinomycetaceae_g_Actinomyces** | **1.592^a^** | **1.772^a^** | **0.035^b^** |
| p_Actinobacteriota_c_Actinobacteria_o_Micrococcales_f_Micrococcaceae_g_Rothia | 0.061 | 0.066 | 0.095 |
| p_Actinobacteriota_c_Coriobacteriia_o_Coriobacteriales_f_Atopobiaceae_g_Atopobium | 0.234 | 0.021 | 0.010 |
| p_Actinobacteriota_c_Coriobacteriia_o_Coriobacteriales_f_Coriobacteriaceae_g_Collinsella | 0.482 | 0.405 | 0.560 |
| p_Actinobacteriota_c_Coriobacteriia_o_Coriobacteriales_f_Eggerthellaceae_g_Eggerthella | 0.071 | 0.076 | 0.004 |
| p_Actinobacteriota_c_Coriobacteriia_o_Coriobacteriales_f_Eggerthellaceae_g_Enterorhabdus | 0.153 | 0.047 | 0.042 |
| p_Actinobacteriota_c_Coriobacteriia_o_Coriobacteriales_f_Eggerthellaceae_g_NA | 0.444 | 0.259 | 0.237 |
| p_Actinobacteriota_c_Coriobacteriia_o_Coriobacteriales_f_Eggerthellaceae_g_Paraeggerthella | 0.460 | 0.443 | 0.072 |
| p_Bacteroidota_c_Bacteroidia_o_Bacteroidales_f_Bacteroidaceae_g_Bacteroides | 4.858 | 4.814 | 2.486 |
| p_Bacteroidota_c_Bacteroidia_o_Bacteroidales_f_Muribaculaceae_g_CAG-873 | 0.493 | 0.259 | 1.116 |
| p_Bacteroidota_c_Bacteroidia_o_Bacteroidales_f_Muribaculaceae_g_NA | 8.127 | 7.113 | 10.967 |
| p_Bacteroidota_c_Bacteroidia_o_Bacteroidales_f_p-2534-18B5 gut group_g_NA | 0.084 | 0.000 | 0.086 |
| p_Bacteroidota_c_Bacteroidia_o_Bacteroidales_f_Prevotellaceae_g_Alloprevotella | 0.107 | 0.097 | 0.477 |
| p_Bacteroidota_c_Bacteroidia_o_Bacteroidales_f_Prevotellaceae_g_NA | 0.162 | 0.000 | 0.000 |
| p_Bacteroidota_c_Bacteroidia_o_Bacteroidales_f_Prevotellaceae_g_Prevotella | 1.629 | 0.943 | 1.987 |
| p_Bacteroidota_c_Bacteroidia_o_Bacteroidales_f_Prevotellaceae_g_Prevotellaceae NK3B31 group | 0.499 | 0.179 | 0.194 |
| p_Bacteroidota_c_Bacteroidia_o_Bacteroidales_f_Prevotellaceae_g_Prevotellaceae UCG-003 | 0.094 | 0.030 | 0.230 |
| p_Bacteroidota_c_Bacteroidia_o_Bacteroidales_f_Rikenellaceae_g_Alistipes | 0.123 | 0.455 | 0.359 |
| **p_Bacteroidota_c_Bacteroidia_o_Bacteroidales_f_Rikenellaceae_g_Rikenellaceae RC9 gut group** | **1.430^a^** | **0.233^b^** | **0.212^b^** |
| p_Bacteroidota_c_Bacteroidia_o_Bacteroidales_f_Tannerellaceae_g_Parabacteroides | 1.160 | 0.643 | 0.236 |
| p_Desulfobacterota_c_Desulfovibrionia_o_Desulfovibrionales_f_Desulfovibrionaceae_g_Desulfovibrio | 0.279 | 0.217 | 0.196 |
| p_Firmicutes_c_Bacilli_o_Erysipelotrichales_f_Erysipelotrichaceae_g_Erysipelotrichaceae UCG-006 | 0.114 | 0.036 | 0.033 |
| p_Firmicutes_c_Bacilli_o_Erysipelotrichales_f_Erysipelotrichaceae_g_Holdemanella | 0.504 | 0.431 | 0.399 |
| p_Firmicutes_c_Bacilli_o_Erysipelotrichales_f_Erysipelotrichaceae_g_NA | 0.121 | 2.225 | 0.196 |
| p_Firmicutes_c_Bacilli_o_Lactobacillales_f_Enterococcaceae_g_Enterococcus | 0.536 | 0.087 | 0.044 |
| p_Firmicutes_c_Bacilli_o_Lactobacillales_f_Lactobacillaceae_g_HT002 | 17.945 | 13.627 | 21.458 |
| **p_Firmicutes_c_Bacilli_o_Lactobacillales_f_Lactobacillaceae_g_Lactobacillus** | **12.143^b^** | **25.352^a^** | **20.673^ab^** |
| **p_Firmicutes_c_Bacilli_o_Lactobacillales_f_Lactobacillaceae_g_Ligilactobacillus** | **0.006^b^** | **0.076^b^** | **0.245^a^** |
| **p_Firmicutes_c_Bacilli_o_Lactobacillales_f_Lactobacillaceae_g_Limosilactobacillus** | **1.223^ab^** | **3.029^a^** | **1.133^b^** |
| p_Firmicutes_c_Bacilli_o_Lactobacillales_f_Streptococcaceae_g_Streptococcus | 2.661 | 2.614 | 1.156 |
| p_Firmicutes_c_Clostridia_o_Christensenellales_f_Christensenellaceae_g_Christensenellaceae R-7 group | 0.195 | 0.335 | 0.183 |
| p_Firmicutes_c_Clostridia_o_Clostridiales_f_Clostridiaceae_g_Clostridium sensu stricto 1 | 3.524 | 2.426 | 2.319 |
| **p_Firmicutes_c_Clostridia_o_Clostridiales_f_Clostridiaceae_g_Clostridium sensu stricto 2** | **0.328^a^** | **0.235^a^** | **0.000^b^** |
| p_Firmicutes_c_Clostridia_o_Lachnospirales_f_Lachnospiraceae_g_[Eubacterium] fissicatena group | 0.904 | 0.531 | 0.686 |
| p_Firmicutes_c_Clostridia_o_Lachnospirales_f_Lachnospiraceae_g_[Eubacterium] hallii group | 0.026 | 0.011 | 0.121 |
| p_Firmicutes_c_Clostridia_o_Lachnospirales_f_Lachnospiraceae_g_[Ruminococcus] gauvreauii group | 0.085 | 0.064 | 0.519 |
| **p_Firmicutes_c_Clostridia_o_Lachnospirales_f_Lachnospiraceae_g_[Ruminococcus] gnavus group** | **0.126^b^** | **0.792^a^** | **0.025^b^** |
| p_Firmicutes_c_Clostridia_o_Lachnospirales_f_Lachnospiraceae_g_[Ruminococcus] torques group | 0.731 | 0.299 | 0.032 |
| p_Firmicutes_c_Clostridia_o_Lachnospirales_f_Lachnospiraceae_g_Blautia | 0.447 | 0.242 | 0.973 |
| p_Firmicutes_c_Clostridia_o_Lachnospirales_f_Lachnospiraceae_g_Dorea | 0.747 | 0.764 | 0.917 |
| p_Firmicutes_c_Clostridia_o_Lachnospirales_f_Lachnospiraceae_g_Eisenbergiella | 0.334 | 0.703 | 0.546 |
| **p_Firmicutes_c_Clostridia_o_Lachnospirales_f_Lachnospiraceae_g_Howardella** | **0.013^b^** | **0.056^a^** | **0.085^a^** |
| p_Firmicutes_c_Clostridia_o_Lachnospirales_f_Lachnospiraceae_g_Hungatella | 0.220 | 0.117 | 0.023 |
| p_Firmicutes_c_Clostridia_o_Lachnospirales_f_Lachnospiraceae_g_Lachnoclostridium | 3.512 | 2.813 | 1.884 |
| p_Firmicutes_c_Clostridia_o_Lachnospirales_f_Lachnospiraceae_g_Lachnospiraceae NK4A136 group | 0.482 | 0.069 | 0.128 |
| p_Firmicutes_c_Clostridia_o_Lachnospirales_f_Lachnospiraceae_g_Marvinbryantia | 0.156 | 0.101 | 0.195 |
| p_Firmicutes_c_Clostridia_o_Lachnospirales_f_Lachnospiraceae_g_NA | 0.650 | 0.156 | 1.313 |
| p_Firmicutes_c_Clostridia_o_Lachnospirales_f_Lachnospiraceae_g_Roseburia | 0.637 | 0.401 | 0.815 |
| p_Firmicutes_c_Clostridia_o_Lachnospirales_f_Lachnospiraceae_g_Tuzzerella | 0.729 | 1.100 | 0.418 |
| p_Firmicutes_c_Clostridia_o_Oscillospirales_f_[Eubacterium] coprostanoligenes group_g_NA | 3.352 | 3.391 | 3.683 |
| p_Firmicutes_c_Clostridia_o_Oscillospirales_f_Butyricicoccaceae_g_Butyricicoccus | 0.121 | 0.146 | 0.493 |
| p_Firmicutes_c_Clostridia_o_Oscillospirales_f_Oscillospiraceae_g_Colidextribacter | 0.720 | 0.471 | 0.940 |
| p_Firmicutes_c_Clostridia_o_Oscillospirales_f_Oscillospiraceae_g_Intestinimonas | 0.660 | 0.165 | 0.203 |
| p_Firmicutes_c_Clostridia_o_Oscillospirales_f_Oscillospiraceae_g_NA | 0.234 | 0.170 | 0.219 |
| p_Firmicutes_c_Clostridia_o_Oscillospirales_f_Oscillospiraceae_g_NK4A214 group | 1.193 | 1.035 | 1.774 |
| p_Firmicutes_c_Clostridia_o_Oscillospirales_f_Oscillospiraceae_g_UCG-002 | 1.543 | 0.762 | 1.604 |
| **p_Firmicutes_c_Clostridia_o_Oscillospirales_f_Oscillospiraceae_g_UCG-005** | **0.157^a^** | **0.072^b^** | **0.226^a^** |
| p_Firmicutes_c_Clostridia_o_Oscillospirales_f_Ruminococcaceae_g_Fournierella | 0.260 | 0.048 | 0.021 |
| p_Firmicutes_c_Clostridia_o_Oscillospirales_f_Ruminococcaceae_g_Incertae Sedis | 0.039 | 0.047 | 0.074 |
| p_Firmicutes_c_Clostridia_o_Oscillospirales_f_Ruminococcaceae_g_NA | 0.294 | 0.324 | 0.478 |
| p_Firmicutes_c_Clostridia_o_Oscillospirales_f_Ruminococcaceae_g_Ruminococcus | 0.733 | 0.585 | 0.261 |
| p_Firmicutes_c_Clostridia_o_Oscillospirales_f_Ruminococcaceae_g_UBA1819 | 0.598 | 0.389 | 0.369 |
| p_Firmicutes_c_Clostridia_o_Oscillospirales_f_UCG-010_g_NA | 0.014 | 0.107 | 0.033 |
| p_Firmicutes_c_Clostridia_o_Peptococcales_f_Peptococcaceae_g_Peptococcus | 0.042 | 0.062 | 0.044 |
| p_Firmicutes_c_Clostridia_o_Peptostreptococcales-Tissierellales_f_Anaerovoracaceae_g_[Eubacterium] brachy group | 0.111 | 0.068 | 0.088 |
| p_Firmicutes_c_Clostridia_o_Peptostreptococcales-Tissierellales_f_Anaerovoracaceae_g_[Eubacterium] nodatum group | 0.769 | 1.242 | 0.295 |
| p_Firmicutes_c_Clostridia_o_Peptostreptococcales-Tissierellales_f_Anaerovoracaceae_g_Family XIII AD3011 group | 0.606 | 0.145 | 0.279 |
| p_Firmicutes_c_Clostridia_o_Peptostreptococcales-Tissierellales_f_Anaerovoracaceae_g_S5-A14a | 0.167 | 0.072 | 0.101 |
| **p_Firmicutes_c_Clostridia_o_Peptostreptococcales-Tissierellales_f_Peptostreptococcaceae_g_Peptostreptococcus** | **0.683^b^** | **1.372^ab^** | **2.010^a^** |
| p_Firmicutes_c_Clostridia_o_Peptostreptococcales-Tissierellales_f_Peptostreptococcaceae_g_Romboutsia | 0.440 | 0.219 | 0.418 |
| p_Firmicutes_c_Clostridia_o_Peptostreptococcales-Tissierellales_f_Peptostreptococcaceae_g_Terrisporobacter | 0.186 | 0.033 | 0.020 |
| **p_Firmicutes_c_Negativicutes_o_Acidaminococcales_f_Acidaminococcaceae_g_Phascolarctobacterium** | **4.509^a^** | **1.077^b^** | **1.196^b^** |
| p_Firmicutes_c_Negativicutes_o_Acidaminococcales_f_Acidaminococcaceae_g_Succiniclasticum | 1.235 | 0.060 | 0.035 |
| p_Firmicutes_c_Negativicutes_o_Veillonellales-Selenomonadales_f_Veillonellaceae_g_Negativicoccus | 0.095 | 0.300 | 0.011 |
| p_Firmicutes_c_Negativicutes_o_Veillonellales-Selenomonadales_f_Veillonellaceae_g_Veillonella | 0.297 | 0.985 | 0.628 |
| p_Fusobacteriota_c_Fusobacteriia_o_Fusobacteriales_f_Fusobacteriaceae_g_Fusobacterium | 1.972 | 3.221 | 3.705 |
| p_NA_c_NA_o_NA_f_NA_g_NA | 2.686 | 0.000 | 0.105 |
| p_Proteobacteria_c_Gammaproteobacteria_o_Burkholderiales_f_Oxalobacteraceae_g_Undibacterium | 0.027 | 0.399 | 0.203 |
| p_Proteobacteria_c_Gammaproteobacteria_o_Enterobacterales_f_Enterobacteriaceae_g_Escherichia-Shigella | 2.603 | 4.977 | 2.547 |
| p_Proteobacteria_c_Gammaproteobacteria_o_Enterobacterales_f_Pasteurellaceae_g_Actinobacillus | 0.009 | 0.126 | 0.219 |
| p_Proteobacteria_c_Gammaproteobacteria_o_Enterobacterales_f_Pasteurellaceae_g_Pasteurella | 0.076 | 0.364 | 0.750 |
| p_Spirochaetota_c_Spirochaetia_o_Spirochaetales_f_Spirochaetaceae_g_Sphaerochaeta | 0.109 | 0.050 | 0.002 |
| **p_Spirochaetota_c_Spirochaetia_o_Spirochaetales_f_Spirochaetaceae_g_Treponema** | **0.520^a^** | **0.000^b^** | **0.117^ab^** |
| **Species** |  |  |  |
| **p_Actinobacteriota_c_Actinobacteria_o_Actinomycetales_f_Actinomycetaceae_g_Actinomyces_s_NA** | **1.592^a^** | **1.772^a^** | **0.035^b^** |
| p_Actinobacteriota_c_Actinobacteria_o_Micrococcales_f_Micrococcaceae_g_Rothia_s_nasimurium | 0.061 | 0.066 | 0.095 |
| p_Actinobacteriota_c_Coriobacteriia_o_Coriobacteriales_f_Atopobiaceae_g_Atopobium_s_minutum | 0.234 | 0.021 | 0.010 |
| p_Actinobacteriota_c_Coriobacteriia_o_Coriobacteriales_f_Coriobacteriaceae_g_Collinsella_s_NA | 0.423 | 0.093 | 0.537 |
| p_Actinobacteriota_c_Coriobacteriia_o_Coriobacteriales_f_Coriobacteriaceae_g_Collinsella_s_tanakaei | 0.060 | 0.311 | 0.022 |
| p_Actinobacteriota_c_Coriobacteriia_o_Coriobacteriales_f_Eggerthellaceae_g_Enterorhabdus_s_NA | 0.153 | 0.047 | 0.042 |
| p_Actinobacteriota_c_Coriobacteriia_o_Coriobacteriales_f_Eggerthellaceae_g_NA_s_NA | 0.444 | 0.259 | 0.237 |
| p_Actinobacteriota_c_Coriobacteriia_o_Coriobacteriales_f_Eggerthellaceae_g_Paraeggerthella_s_NA | 0.460 | 0.443 | 0.072 |
| p_Bacteroidota_c_Bacteroidia_o_Bacteroidales_f_Bacteroidaceae_g_Bacteroides_s_fluxus | 0.001 | 0.595 | 0.111 |
| p_Bacteroidota_c_Bacteroidia_o_Bacteroidales_f_Bacteroidaceae_g_Bacteroides_s_fragilis | 1.292 | 0.624 | 0.757 |
| p_Bacteroidota_c_Bacteroidia_o_Bacteroidales_f_Bacteroidaceae_g_Bacteroides_s_heparinolyticus | 0.419 | 0.352 | 0.031 |
| p_Bacteroidota_c_Bacteroidia_o_Bacteroidales_f_Bacteroidaceae_g_Bacteroides_s_NA | 0.198 | 0.494 | 0.014 |
| p_Bacteroidota_c_Bacteroidia_o_Bacteroidales_f_Bacteroidaceae_g_Bacteroides_s_plebeius | 0.116 | 0.170 | 0.084 |
| p_Bacteroidota_c_Bacteroidia_o_Bacteroidales_f_Bacteroidaceae_g_Bacteroides_s_pyogenes | 0.885 | 0.885 | 0.169 |
| p_Bacteroidota_c_Bacteroidia_o_Bacteroidales_f_Bacteroidaceae_g_Bacteroides_s_thetaiotaomicron | 0.714 | 0.258 | 0.085 |
| **p_Bacteroidota_c_Bacteroidia_o_Bacteroidales_f_Bacteroidaceae_g_Bacteroides_s_uniformis** | **0.109^a^** | **0.061^ab^** | **0.040^b^** |
| p_Bacteroidota_c_Bacteroidia_o_Bacteroidales_f_Bacteroidaceae_g_Bacteroides_s_vulgatus | 0.858 | 1.014 | 1.070 |
| p_Bacteroidota_c_Bacteroidia_o_Bacteroidales_f_Bacteroidaceae_g_Bacteroides_s_xylanisolvens | 0.142 | 0.149 | 0.014 |
| p_Bacteroidota_c_Bacteroidia_o_Bacteroidales_f_Muribaculaceae_g_CAG-873_s_NA | 0.493 | 0.259 | 1.116 |
| p_Bacteroidota_c_Bacteroidia_o_Bacteroidales_f_Muribaculaceae_g_NA_s_NA | 8.127 | 7.113 | 10.967 |
| p_Bacteroidota_c_Bacteroidia_o_Bacteroidales_f_p-2534-18B5 gut group_g_NA_s_NA | 0.084 | 0.000 | 0.086 |
| p_Bacteroidota_c_Bacteroidia_o_Bacteroidales_f_Prevotellaceae_g_Alloprevotella_s_NA | 0.107 | 0.097 | 0.477 |
| p_Bacteroidota_c_Bacteroidia_o_Bacteroidales_f_Prevotellaceae_g_NA_s_NA | 0.162 | 0.000 | 0.000 |
| p_Bacteroidota_c_Bacteroidia_o_Bacteroidales_f_Prevotellaceae_g_Prevotella_s_NA | 1.362 | 0.897 | 1.443 |
| p_Bacteroidota_c_Bacteroidia_o_Bacteroidales_f_Prevotellaceae_g_Prevotella_s_stercorea | 0.256 | 0.022 | 0.544 |
| p_Bacteroidota_c_Bacteroidia_o_Bacteroidales_f_Prevotellaceae_g_Prevotellaceae NK3B31 group_s_NA | 0.499 | 0.179 | 0.194 |
| p_Bacteroidota_c_Bacteroidia_o_Bacteroidales_f_Prevotellaceae_g_Prevotellaceae UCG-003_s_NA | 0.094 | 0.030 | 0.230 |
| p_Bacteroidota_c_Bacteroidia_o_Bacteroidales_f_Rikenellaceae_g_Alistipes_s_NA | 0.022 | 0.136 | 0.007 |
| p_Bacteroidota_c_Bacteroidia_o_Bacteroidales_f_Rikenellaceae_g_Alistipes_s_shahii | 0.050 | 0.319 | 0.346 |
| **p_Bacteroidota_c_Bacteroidia_o_Bacteroidales_f_Rikenellaceae_g_Rikenellaceae RC9 gut group_s_NA** | **1.430^a^** | **0.233^b^** | **0.212^b^** |
| p_Bacteroidota_c_Bacteroidia_o_Bacteroidales_f_Tannerellaceae_g_Parabacteroides_s_distasonis | 0.826 | 0.225 | 0.185 |
| p_Bacteroidota_c_Bacteroidia_o_Bacteroidales_f_Tannerellaceae_g_Parabacteroides_s_merdae | 0.334 | 0.418 | 0.051 |
| p_Desulfobacterota_c_Desulfovibrionia_o_Desulfovibrionales_f_Desulfovibrionaceae_g_Desulfovibrio_s_piger | 0.279 | 0.217 | 0.196 |
| p_Firmicutes_c_Bacilli_o_Erysipelotrichales_f_Erysipelotrichaceae_g_Erysipelotrichaceae UCG-006_s_NA | 0.114 | 0.036 | 0.033 |
| p_Firmicutes_c_Bacilli_o_Erysipelotrichales_f_Erysipelotrichaceae_g_Holdemanella_s_NA | 0.504 | 0.431 | 0.399 |
| p_Firmicutes_c_Bacilli_o_Erysipelotrichales_f_Erysipelotrichaceae_g_NA_s_NA | 0.121 | 2.225 | 0.196 |
| p_Firmicutes_c_Bacilli_o_Lactobacillales_f_Enterococcaceae_g_Enterococcus_s_faecium | 0.510 | 0.047 | 0.041 |
| p_Firmicutes_c_Bacilli_o_Lactobacillales_f_Lactobacillaceae_g_HT002_s_NA | 17.945 | 13.627 | 21.458 |
| p_Firmicutes_c_Bacilli_o_Lactobacillales_f_Lactobacillaceae_g_Lactobacillus_s_amylovorus | 5.239 | 5.643 | 5.113 |
| **p_Firmicutes_c_Bacilli_o_Lactobacillales_f_Lactobacillaceae_g_Lactobacillus_s_delbrueckii** | **3.777^b^** | **12.140^a^** | **6.961^ab^** |
| **p_Firmicutes_c_Bacilli_o_Lactobacillales_f_Lactobacillaceae_g_Lactobacillus_s_johnsonii** | **2.686^b^** | **6.607^a^** | **5.562^a^** |
| p_Firmicutes_c_Bacilli_o_Lactobacillales_f_Lactobacillaceae_g_Lactobacillus_s_NA | 0.441 | 0.963 | 3.037 |
| **p_Firmicutes_c_Bacilli_o_Lactobacillales_f_Lactobacillaceae_g_Ligilactobacillus_s_NA** | **0.006^b^** | **0.076^b^** | **0.245^a^** |
| **p_Firmicutes_c_Bacilli_o_Lactobacillales_f_Lactobacillaceae_g_Limosilactobacillus_s_NA** | **1.223^ab^** | **3.029^a^** | **1.133^b^** |
| p_Firmicutes_c_Bacilli_o_Lactobacillales_f_Streptococcaceae_g_Streptococcus_s_gallolyticus | 0.733 | 1.291 | 1.041 |
| **p_Firmicutes_c_Bacilli_o_Lactobacillales_f_Streptococcaceae_g_Streptococcus_s_hyointestinalis** | **0.294^b^** | **0.907^a^** | **0.046^b^** |
| p_Firmicutes_c_Bacilli_o_Lactobacillales_f_Streptococcaceae_g_Streptococcus_s_lutetiensis | 1.546 | 0.069 | 0.000 |
| p_Firmicutes_c_Bacilli_o_Lactobacillales_f_Streptococcaceae_g_Streptococcus_s_suis | 0.047 | 0.251 | 0.025 |
| p_Firmicutes_c_Clostridia_o_Christensenellales_f_Christensenellaceae_g_Christensenellaceae R-7 group_s_NA | 0.195 | 0.335 | 0.183 |
| p_Firmicutes_c_Clostridia_o_Clostridiales_f_Clostridiaceae_g_Clostridium sensu stricto 1_s_NA | 0.763 | 0.307 | 0.329 |
| p_Firmicutes_c_Clostridia_o_Clostridiales_f_Clostridiaceae_g_Clostridium sensu stricto 1_s_perfringens | 2.698 | 2.119 | 1.946 |
| **p_Firmicutes_c_Clostridia_o_Clostridiales_f_Clostridiaceae_g_Clostridium sensu stricto 2_s_NA** | **0.328^a^** | **0.235^a^** | **0.000^b^** |
| p_Firmicutes_c_Clostridia_o_Lachnospirales_f_Lachnospiraceae_g_[Eubacterium] fissicatena group_s_NA | 0.904 | 0.531 | 0.686 |
| p_Firmicutes_c_Clostridia_o_Lachnospirales_f_Lachnospiraceae_g_[Ruminococcus] gauvreauii group_s_NA | 0.085 | 0.064 | 0.519 |
| **p_Firmicutes_c_Clostridia_o_Lachnospirales_f_Lachnospiraceae_g_[Ruminococcus] gnavus group_s_NA** | **0.118^b^** | **0.742^a^** | **0.025^b^** |
| p_Firmicutes_c_Clostridia_o_Lachnospirales_f_Lachnospiraceae_g_[Ruminococcus] torques group_s_NA | 0.041 | 0.278 | 0.032 |
| p_Firmicutes_c_Clostridia_o_Lachnospirales_f_Lachnospiraceae_g_[Ruminococcus] torques group_s_torques | 0.691 | 0.021 | 0.000 |
| p_Firmicutes_c_Clostridia_o_Lachnospirales_f_Lachnospiraceae_g_Blautia_s_hansenii | 0.087 | 0.061 | 0.043 |
| p_Firmicutes_c_Clostridia_o_Lachnospirales_f_Lachnospiraceae_g_Blautia_s_NA | 0.315 | 0.172 | 0.849 |
| p_Firmicutes_c_Clostridia_o_Lachnospirales_f_Lachnospiraceae_g_Dorea_s_NA | 0.747 | 0.764 | 0.917 |
| p_Firmicutes_c_Clostridia_o_Lachnospirales_f_Lachnospiraceae_g_Eisenbergiella_s_NA | 0.334 | 0.703 | 0.546 |
| **p_Firmicutes_c_Clostridia_o_Lachnospirales_f_Lachnospiraceae_g_Howardella_s_NA** | **0.013^b^** | **0.056^a^** | **0.085^a^** |
| p_Firmicutes_c_Clostridia_o_Lachnospirales_f_Lachnospiraceae_g_Hungatella_s_NA | 0.220 | 0.117 | 0.023 |
| p_Firmicutes_c_Clostridia_o_Lachnospirales_f_Lachnospiraceae_g_Lachnoclostridium_s_NA | 3.512 | 2.813 | 1.884 |
| p_Firmicutes_c_Clostridia_o_Lachnospirales_f_Lachnospiraceae_g_Lachnospiraceae NK4A136 group_s_NA | 0.482 | 0.069 | 0.128 |
| p_Firmicutes_c_Clostridia_o_Lachnospirales_f_Lachnospiraceae_g_Marvinbryantia_s_NA | 0.156 | 0.101 | 0.195 |
| p_Firmicutes_c_Clostridia_o_Lachnospirales_f_Lachnospiraceae_g_NA_s_NA | 0.650 | 0.156 | 1.313 |
| p_Firmicutes_c_Clostridia_o_Lachnospirales_f_Lachnospiraceae_g_Roseburia_s_inulinivorans | 0.552 | 0.391 | 0.803 |
| p_Firmicutes_c_Clostridia_o_Lachnospirales_f_Lachnospiraceae_g_Tuzzerella_s_NA | 0.729 | 1.100 | 0.418 |
| p_Firmicutes_c_Clostridia_o_Oscillospirales_f_[Eubacterium] coprostanoligenes group_g_NA_s_NA | 3.352 | 3.391 | 3.683 |
| p_Firmicutes_c_Clostridia_o_Oscillospirales_f_Butyricicoccaceae_g_Butyricicoccus_s_NA | 0.064 | 0.066 | 0.379 |
| p_Firmicutes_c_Clostridia_o_Oscillospirales_f_Butyricicoccaceae_g_Butyricicoccus_s_pullicaecorum | 0.056 | 0.080 | 0.114 |
| p_Firmicutes_c_Clostridia_o_Oscillospirales_f_Oscillospiraceae_g_Colidextribacter_s_NA | 0.720 | 0.471 | 0.940 |
| **p_Firmicutes_c_Clostridia_o_Oscillospirales_f_Oscillospiraceae_g_Intestinimonas_s_butyriciproducens** | **0.103^a^** | **0.011^b^** | **0.038^ab^** |
| p_Firmicutes_c_Clostridia_o_Oscillospirales_f_Oscillospiraceae_g_Intestinimonas_s_NA | 0.557 | 0.153 | 0.164 |
| p_Firmicutes_c_Clostridia_o_Oscillospirales_f_Oscillospiraceae_g_NA_s_NA | 0.234 | 0.170 | 0.219 |
| p_Firmicutes_c_Clostridia_o_Oscillospirales_f_Oscillospiraceae_g_NK4A214 group_s_NA | 1.193 | 1.035 | 1.774 |
| p_Firmicutes_c_Clostridia_o_Oscillospirales_f_Oscillospiraceae_g_UCG-002_s_NA | 1.543 | 0.762 | 1.604 |
| **p_Firmicutes_c_Clostridia_o_Oscillospirales_f_Oscillospiraceae_g_UCG-005_s_NA** | **0.157^a^** | **0.072^b^** | **0.226^a^** |
| p_Firmicutes_c_Clostridia_o_Oscillospirales_f_Ruminococcaceae_g_Fournierella_s_NA | 0.260 | 0.048 | 0.021 |
| p_Firmicutes_c_Clostridia_o_Oscillospirales_f_Ruminococcaceae_g_Incertae Sedis_s_NA | 0.039 | 0.047 | 0.074 |
| p_Firmicutes_c_Clostridia_o_Oscillospirales_f_Ruminococcaceae_g_NA_s_NA | 0.294 | 0.324 | 0.478 |
| p_Firmicutes_c_Clostridia_o_Oscillospirales_f_Ruminococcaceae_g_Ruminococcus_s_NA | 0.733 | 0.585 | 0.261 |
| p_Firmicutes_c_Clostridia_o_Oscillospirales_f_Ruminococcaceae_g_UBA1819_s_NA | 0.598 | 0.389 | 0.369 |
| p_Firmicutes_c_Clostridia_o_Oscillospirales_f_UCG-010_g_NA_s_NA | 0.014 | 0.107 | 0.033 |
| p_Firmicutes_c_Clostridia_o_Peptococcales_f_Peptococcaceae_g_Peptococcus_s_NA | 0.042 | 0.062 | 0.044 |
| p_Firmicutes_c_Clostridia_o_Peptostreptococcales-Tissierellales_f_Anaerovoracaceae_g_[Eubacterium] brachy group_s_NA | 0.111 | 0.068 | 0.088 |
| p_Firmicutes_c_Clostridia_o_Peptostreptococcales-Tissierellales_f_Anaerovoracaceae_g_[Eubacterium] nodatum group_s_NA | 0.702 | 0.901 | 0.295 |
| p_Firmicutes_c_Clostridia_o_Peptostreptococcales-Tissierellales_f_Anaerovoracaceae_g_[Eubacterium] nodatum group_s_nodatum | 0.000 | 0.307 | 0.000 |
| p_Firmicutes_c_Clostridia_o_Peptostreptococcales-Tissierellales_f_Anaerovoracaceae_g_Family XIII AD3011 group_s_NA | 0.606 | 0.145 | 0.279 |
| p_Firmicutes_c_Clostridia_o_Peptostreptococcales-Tissierellales_f_Anaerovoracaceae_g_S5-A14a_s_NA | 0.167 | 0.072 | 0.101 |
| **p_Firmicutes_c_Clostridia_o_Peptostreptococcales-Tissierellales_f_Peptostreptococcaceae_g_Peptostreptococcus_s_NA** | **0.683^b^** | **1.372^ab^** | **2.010^a^** |
| p_Firmicutes_c_Clostridia_o_Peptostreptococcales-Tissierellales_f_Peptostreptococcaceae_g_Romboutsia_s_ilealis | 0.440 | 0.219 | 0.418 |
| p_Firmicutes_c_Clostridia_o_Peptostreptococcales-Tissierellales_f_Peptostreptococcaceae_g_Terrisporobacter_s_NA | 0.186 | 0.033 | 0.020 |
| **p_Firmicutes_c_Negativicutes_o_Acidaminococcales_f_Acidaminococcaceae_g_Phascolarctobacterium_s_faecium** | **0.001^b^** | **0.013^b^** | **0.148^a^** |
| **p_Firmicutes_c_Negativicutes_o_Acidaminococcales_f_Acidaminococcaceae_g_Phascolarctobacterium_s_NA** | **4.508^a^** | **1.064^b^** | **1.048^b^** |
| p_Firmicutes_c_Negativicutes_o_Acidaminococcales_f_Acidaminococcaceae_g_Succiniclasticum_s_NA | 1.235 | 0.060 | 0.035 |
| p_Firmicutes_c_Negativicutes_o_Veillonellales-Selenomonadales_f_Veillonellaceae_g_Negativicoccus_s_NA | 0.095 | 0.300 | 0.011 |
| p_Firmicutes_c_Negativicutes_o_Veillonellales-Selenomonadales_f_Veillonellaceae_g_Veillonella_s_caviae | 0.297 | 0.931 | 0.623 |
| p_Fusobacteriota_c_Fusobacteriia_o_Fusobacteriales_f_Fusobacteriaceae_g_Fusobacterium_s_gastrosuis | 1.091 | 1.296 | 0.896 |
| p_Fusobacteriota_c_Fusobacteriia_o_Fusobacteriales_f_Fusobacteriaceae_g_Fusobacterium_s_mortiferum | 0.421 | 0.759 | 2.537 |
| p_Fusobacteriota_c_Fusobacteriia_o_Fusobacteriales_f_Fusobacteriaceae_g_Fusobacterium_s_NA | 0.259 | 0.102 | 0.072 |
| p_Fusobacteriota_c_Fusobacteriia_o_Fusobacteriales_f_Fusobacteriaceae_g_Fusobacterium_s_varium | 0.149 | 0.988 | 0.054 |
| p_NA_c_NA_o_NA_f_NA_g_NA_s_NA | 2.686 | 0.000 | 0.105 |
| p_Proteobacteria_c_Gammaproteobacteria_o_Burkholderiales_f_Oxalobacteraceae_g_Undibacterium_s_NA | 0.027 | 0.399 | 0.203 |
| p_Proteobacteria_c_Gammaproteobacteria_o_Enterobacterales_f_Enterobacteriaceae_g_Escherichia-Shigella_s_albertii | 0.008 | 0.080 | 0.742 |
| p_Proteobacteria_c_Gammaproteobacteria_o_Enterobacterales_f_Enterobacteriaceae_g_Escherichia-Shigella_s_coli | 2.595 | 4.726 | 1.454 |
| p_Proteobacteria_c_Gammaproteobacteria_o_Enterobacterales_f_Enterobacteriaceae_g_Escherichia-Shigella_s_NA | 0.000 | 0.170 | 0.351 |
| p_Proteobacteria_c_Gammaproteobacteria_o_Enterobacterales_f_Pasteurellaceae_g_Actinobacillus_s_indolicus | 0.000 | 0.122 | 0.053 |
| **p_Proteobacteria_c_Gammaproteobacteria_o_Enterobacterales_f_Pasteurellaceae_g_Pasteurella_s_aerogenes** | **0.076^b^** | **0.364^ab^** | **0.750^a^** |
| p_Spirochaetota_c_Spirochaetia_o_Spirochaetales_f_Spirochaetaceae_g_Sphaerochaeta_s_NA | 0.109 | 0.050 | 0.002 |
| **p_Spirochaetota_c_Spirochaetia_o_Spirochaetales_f_Spirochaetaceae_g_Treponema_s_brennaborense** | **0.398^a^** | **0.000^b^** | **0.117^ab^** |

^a^ Means within row without common superscript are significantly different, P<0.05. CTRL: control group; Low scFOS: sows supplemented with 0.15 % scFOS from d28 pre-partum to d2 post-partum (gestation and transition diet) and 0.10 % during lactation (lactation diet); Mid scFOS: sows supplemented with 0.33 % scFOS from d28 pre-partum to d2 post-partum (gestation and transition diet) and 0.15 % during lactation (lactation diet).
